# A Soil Bulk Density Metric to Improve Rapid Assessment of Wetland Condition

**DOI:** 10.1007/s13157-026-02044-9

**Published:** 2026-07-07

**Authors:** Katie Hossler, James Tyler Retherford, Mitchell Link

**Affiliations:** Department of Biological Sciences, Wright State University, 3640 Colonel Glenn Hwy, Dayton, OH 45435 USA

**Keywords:** Wetland assessment, Wetland quality, Soil bulk density, Soil health

## Abstract

Rapid wetland assessments are designed to require minimal field effort (e.g. half-day site visit), but still provide critical information on wetland ecological condition and functioning. Many wetland functions occur within or are supported by the soil, yet soil condition is seldom–or only minimally–included in most state rapid assessments. We focus on the Ohio Rapid Assessment Method for wetlands (ORAM) and evaluate soil bulk density as an indicator of wetland soil condition that can be incorporated into rapid wetland assessments. Soil bulk density ($\boldsymbol{\rho }_{\boldsymbol{b}}$) is relatively easy to measure and requires only basic instrumentation. It also correlates well with more intensive metrics of soil condition (e.g. carbon content, aggregation). We sampled 45 wetlands in west–central Ohio, that exhibited a range of wetland type and ecological condition. From each wetland, we collected five soil cores (to 15-cm depth) and then analyzed the soil samples for $\boldsymbol{\rho }_{\boldsymbol{b}}$, carbon content, and water-stable aggregates. These three measurements were used to develop a four-bin $\boldsymbol{\rho }_{\boldsymbol{b}}$ metric of soil condition. Incorporating this metric into the current ORAM improved relationships with $\boldsymbol{\rho }_{\boldsymbol{b}}$ (*r* = 0.48 to *r* = 0.58) and soil condition (*r* = -0.53 to *r* = -0.64). Additional gains could be achieved with higher weighting of the $\boldsymbol{\rho }_{\boldsymbol{b}}$ metric. Alternatively, the current scoring system could be left intact, but protection of wetlands with high quality soils ensured by providing special status when $\boldsymbol{\rho }_{\boldsymbol{b}}$ is less than 0.40 g cm$^{\boldsymbol{-3}}$ (highest quality) or less than 0.73 g cm$^{\boldsymbol{-3}}$ (high quality).

**Supplementary Information:**

The online version contains supplementary material available at https://doi.org/10.1007/s13157-026-02044-9.

## Introduction

The soil is the foundation for plant growth and microbial activity, which collectively support key ecosystem functions such as biodiversity and biogeochemical cycling. Despite the importance of soil, it is seldom included as a metric in Level 2 (“rapid”) assessments of wetland condition. In Ohio, for example, wetland condition is primarily assessed with metrics related to size and surrounding land use (20% contribution to total score), hydrology (30%), and vegetation (20%) (Mack 2001a). The soil condition is included only cursorily through a submetric rating substrate disturbance (4%). The omission or superficial treatment of soil attributes in Level 2 wetland assessments is not unique to Ohio; see for example, the review by Fennessy et al. (2007). More recently, Faber-Langendoen et al. (2019), developed a rapid assessment protocol for evaluating the ecological integrity of wetlands across the nation. Their protocol does include one soil metric (out of 12 recommended metrics total); however, the metric assesses only surficial substrate disturbance (see also Faber-Langendoen et al. 2016) and contributes only 7 % to the final assessment score.

In contrast, principal soil functions are carried out throughout the soil profile. The physical structure of the soil throughout the profile determines the extent of functions such as storage of carbon and nutrients, transport and storage of water, and support for plant growth and microbial activity (Elliott and Coleman 1988; Carter 2002; Raghavendra et al. 2020). Good soil structure—e.g. highly aggregated, low bulk density—promotes these functions. Bulk density, in particular, is one attribute of soil structure that correlates well with multiple indicators of soil and wetland function and is fairly easy to measure (Rokosch et al. 2009; Hossler et al. 2011; Fennessy and Wardrop 2016; Onufrak et al. 2020). Herein we develop and evaluate a bulk density based metric to be included in current rapid assessments of wetland condition. Inclusion of the soil based metric should better reflect the condition of the soil and thus overall wetland condition.

### Relevance

Wetlands provide many key ecosystem services (e.g. support for biodiversity, flood protection, nutrient cycling), yet over half of the original wetland base in the conterminous United States has been lost since European settlement (Dahl 1990). In growing recognition of the importance of wetlands, however, protections are now provided under the Clean Water Act. Under this policy, impacts to wetlands are regulated typically by authorized states. To prioritize wetland protections (e.g. preserve highest quality/functioning wetlands), many states rely on assessment methods. In particular, Level 2 or “rapid” assessments are utilized for coarse assessment of ecological condition with minimal effort (e.g. half-day site visit).

We focused on the Level 2 assessment for Ohio or Ohio Rapid Assessment Method (ORAM). The ORAM was modeled after a rapid assessment method developed by the State of Washington (State of Washington 1993) with some modification (Fennessy et al. 1998; Mack 2001a). The most recent version (ORAM v5.0; Mack 2001a) is comprised of six metrics: wetland size (6%), surrounding land use (14%), hydrology (30%), habitat alteration (20%), special wetland communities (10%), and vegetation (20%). These metrics are combined into a single score that reflects the ecological functions provided by a wetland relative to an unimpaired “reference” wetland of similar type. The ORAM has been demonstrated to be a reliable indicator of disturbance (*n=21*, $R^2=0.66$), vegetation quality (*n=13*, $R^2=0.76$), other biological indicators such as macroinvertebrate taxa richness (emergent wetlands only, *n=7*, $R^2=0.92$), and a vegetation-based index of biological integrity (*n=49*, $R^2 = 0.84$) (Fennessy et al. 1998; Mack et al. 2000).

One key wetland component missing from the ORAM methodology and validation is the soil. This is concerning given that many wetland functions occur within or are supported by the soil (e.g. primary production, carbon sequestration, nutrient cycling). Two recent studies have evaluated the ORAM with respect to various soil-based metrics (Rokosch et al. 2009; Onufrak et al. 2020). Both studies were small in scope but suggested that although the ORAM correlated with some soil parameters, the relationship could be improved. The Rokosch et al. (2009) study of six forested depressional wetlands, for example, observed a correlation between ORAM score and soil parameters such as carbon, nitrogen and microbial biomass, but soil parameters indicating aggregation (a key soil structural property) were uncorrelated. Onufrak et al. (2020) assessed soil fungal communities in six emergent depressional wetlands and observed a relationship between ORAM and fungal community composition, but not between ORAM and fungal diversity or richness, or fungal functional guild composition.

Both Rokosch et al. (2009) and Onufrak et al. (2020) recommended that one soil property in particular—soil bulk density—be included in wetland assessments because of its strong correlation to multiple physical, chemical and biological soil parameters and ease of measurement. Gara and Schumacher (2015), in their report of wetland condition in Ohio, recommended that soil structure (albeit not necessarily bulk density) be included in future wetland assessments. Other wetland studies have also noted the utility of soil bulk density as a readily quantifiable metric to monitor wetlands. Meyer et al. (2008) found soil bulk density (and soil organic matter) to be a good index of recovery for 6 degraded wet meadows. Hossler et al. (2011), in their study of 5 natural and 10 created wetlands, found that soil bulk density was a good indicator of multiple soil metrics (e.g. soil carbon, microbial biomass) and wetland functions facilitated by the soil (e.g. primary production, carbon mineralization, denitrification).

In the broader literature, soil bulk density—in conjunction with other soil properties—is recommended in assessments of soil condition in response to land management practices (e.g. Doran and Parkin 1996; Schoenholtz et al. 2000; Maurya et al. 2020). Ideally, we would assess multiple soil properties, in addition to bulk density, to better assess soil condition (Doran and Parkin 1996; Smith and Klimas 2002; Maurya et al. 2020); however, we focus on bulk density because it fits better into the “rapid assessment” framework. While measurement of many important soil properties requires specialized equipment or lengthy procedures (e.g. soil carbon, aggregation, microbial community), assessment of soil bulk density—or the dry mass relative to field-moist volume—requires only a drying oven, scale and means to quantify soil volume (e.g. corer) and is relatively low effort to obtain. Soil bulk density is also responsive to change (Hossler and Bouchard 2010; Or et al. 2021)—another criterion for an effective indicator (Doran and Parkin 1996; Smith et al. 2013; Fennessy and Wardrop 2016).

The primary objective of this study was to improve the relationship of the current ORAM to overall soil health, by incorporating measurement of soil bulk density—a proxy for soil health—into the ORAM. We sampled 45 wetlands throughout Ohio that represented a range of wetland types and “quality.” In addition to soil bulk density, we measured soil carbon (a key metric for soil chemistry and composition) and soil aggregation (a key metric for soil structure) to provide additional assessment of soil condition; however, our focus was on soil bulk density as the basis for a new ORAM metric.

The project had five aims:

1. Assess soil health using measurements of soil bulk density, carbon content, and aggregation
2. Evaluate the relationship between the current ORAM (v5.0) and soil health
3. Verify the suitability of soil bulk density as a proxy for soil health
4. Develop a bulk density based metric to include in the ORAM
5. Evaluate the relationship of a revised ORAM (v6.0) to soil health.

The revised ORAM (v6.0) should better reflect the overall wetland condition, as it will now be based on aspects of hydrology, vegetation, *and soil*. This more holistic assessment will improve detection and prioritization of high-quality wetlands with respect to all three requisite wetland components (i.e. hydrology, vegetation, soil; Environmental Laboratory 1987; Federal Interagency Committee for Wetland Delineation 1989).

## Methods

### Site Selection

Our sample set consisted of 45 wetlands throughout west–central Ohio that represented a range of wetland types and ecological condition or “quality.” Wetland quality was based on ORAM v5.0 (Mack 2001a) which included both a score (0–100) and a category (1–3). The ORAM metrics and submetrics scored wetlands based on function (e.g. hydrologic connectivity), anthropogenic impacts to those functions (e.g. hydrologic modification), and features that added particular value (e.g. some wetlands in Ohio receive extra points for being in certain regions). Thus, “high quality” wetlands (Category 3, ORAM score *≥ 60*) were typically high functioning wetlands with little human-caused degradation, whereas “low quality” wetlands (Category 1, ORAM score *< 30*) were low functioning wetlands, often because of human-caused degradation. “Medium quality” wetlands (Category 2, ORAM score *≥ 30* and *< 60*) provided moderate levels of function, which can be inherent to the wetland or because of human-caused degradation (Mack 2001a).

The ORAM has been demonstrated to be relatively robust across different wetland hydrogeomorphic (HGM) and vegetation classes, and across regions in Ohio (Mack 2001b). Soil condition, however, might be sensitive to HGM and vegetation class given that topography and organisms are two of the main soil forming factors (Jenny 1941; see also Noble and Berkowitz 2016; Daugherty et al. 2019). Thus, in addition to obtaining a representative sample of wetland condition (based on ORAM score and category), we also wanted a representative sample across HGM classes (specifically depression, riverine and slope) and vegetation classes (emergent, shrub and forest). We targeted six HGM–vegetation subgroups: depression emergent, depression forest, depression shrub, riverine emergent, riverine forest, slope emergent.

**Fig. 1:**
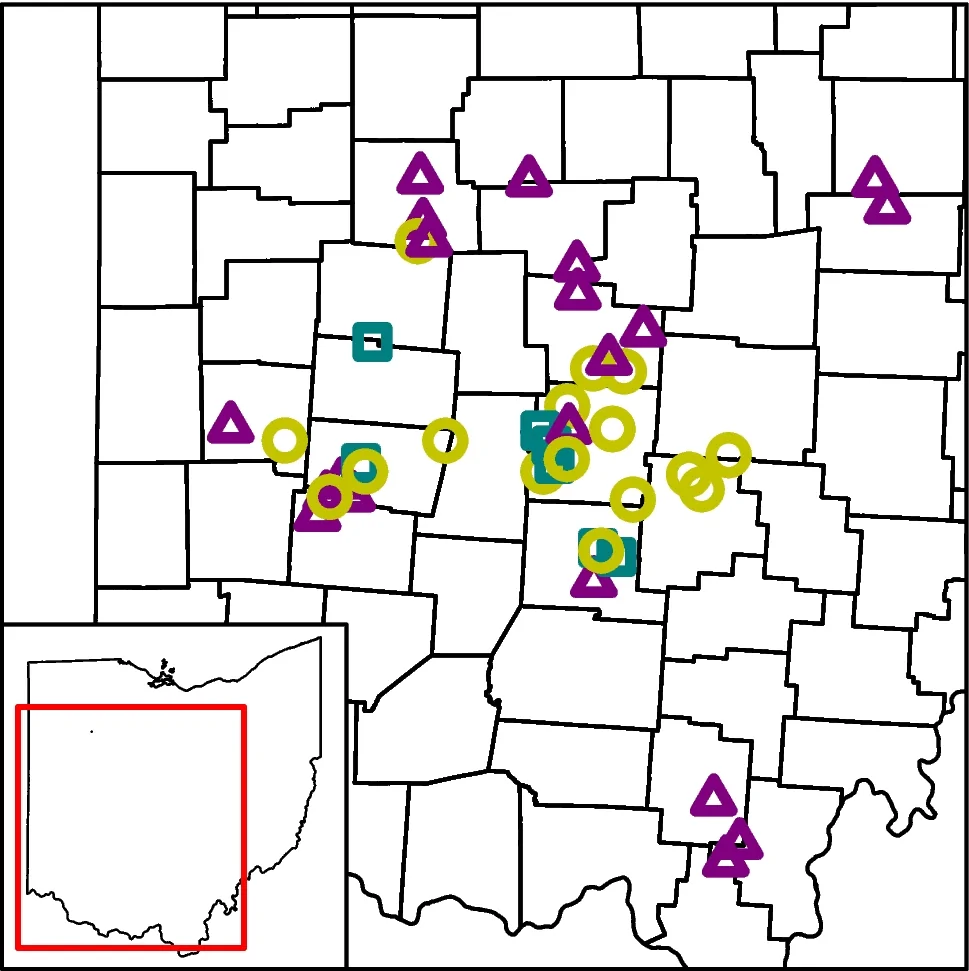
Map of the 45 wetlands across west-central Ohio that were sampled. The inset shows the region of Ohio. Wetlands are shaped and colored by ORAM v5.0 category: green squares, Category 1; yellow circles, Category 2; purple triangles, Category 3. The three ORAM categories refer to wetland ecological condition or quality: low, medium, high, respectively. See also “Site Selection,” Table 1, and Supplementary Section S2

There were 256 potential wetland sites that previously had been scored using the ORAM (primarily by the Ohio EPA), that were in the target region of west–central Ohio, and belonged to the six targeted HGM-vegetation subgroups. We selected 11 of the sites by purposive sampling, as “legacy” or “special interest” sites. 34 sites were selected randomly using a Generalized Random Tessellation Stratified survey design for a finite resource (Stevens and Olsen 1999, 2004). This methodology has been used in other wetland surveys (USEPA 2016; Gara and Schumacher 2015; Olsen et al. 2019), and helps achieve a geographically-balanced random sample with additional constraints such as representation across ORAM category and HGM-vegetation subgroup (Stevens and Jensen 2007; Lackey and Stein 2013; see also Supplementary Section S1). The 45 sites comprising the final sample set spanned 16 counties in west–central Ohio (Fig. 1).

Many of the ORAM scores were outdated: e.g. most wetlands were scored over ten years ago, with some scored over twenty years ago. We updated all wetland ORAM scores and categories using aerial imagery and notes from our field sampling. Additional details are provided in Supplementary Section S2. With the re-assignment of ORAM categories, the distribution of wetlands across the three categories shifted (Table 1). The distribution of wetlands based on ORAM score, however, remained the same: i.e. ORAM score mean and range were similar between the original and current assessments.

**Table 1:** Representation of the three ORAM categories and six HGM-vegetation subgroups across the 45 wetlands (for definitions see “Site Selection”). The numbers in the table refer to the number of wetlands belonging to a particular ORAM category (after re-assessment) and HGM-vegetation subgroup. Re-assessment of ORAM scores resulted in the upgrading of five formerly Category 1 wetlands and six formerly Category 2 wetlands, which skewed the sample set to the higher quality categories

	ORAM Category			
HGM-vegetation	Cat 1	Cat 2	Cat 3	Total
Depression-emergent	4	3	2	9
Depression-forest	1	2	4	7
Depression-shrub	0	3	3	6
Riverine-emergent	2	5	2	9
Riverine-forest	0	3	3	6
Slope-emergent	0	1	7	8
Total	7	17	21	45

### Soil Sample Collection and Analyses

Soil samples were collected using a modified stratified random sampling strategy (additional details in Supplementary Section S3). Our sampling strategy for sample location balanced ease of sample collection, spatial representation for a limited number of samples per wetland, and inference strength. Spatial representation is of particular importance because soil properties can exhibit high spatial variability (Doran and Parkin 1996; Smith and Klimas 2002). In brief, five soil samples were collected per wetland, targeting five a priori elevational zones. The zones were based on elevational gradients present in most wetlands. These elevational gradients are known to create zones with characteristic hydrology, vegetation, and soil (Stewart and Kantrud 1971; Millar 1976; Cowardin et al. 1979; Reinecke et al. 2015; Daugherty et al. 2019). Stewart and Kantrud (1971), for example, identified up to five zones within a wetland that had unique hydroperiods and vegetation communities.

Prior to each site visit, we first delineated wetland boundaries using recent and historical imagery available through Google Earth. When possible, these boundaries were based on National Wetland Inventory (NWI) polygon shapefiles. Wetlands were assumed to have either edge-to-interior gradients (e.g. all depressional and some slope wetlands) or upland-to-stream gradients (e.g. all riverine and some slope wetlands). A transect pair was randomly selected within each wetland, resulting in either two perpendicular radial transects (i.e. edge-to-interior gradients, Supplementary Fig. S1a,b; see also Supplementary Section S4 and Supplementary Figs. S2–S4) or two parallel transects (i.e. upland-to-stream gradients, Supplementary Fig. S1c,d). Each transect intersected all five elevational zones. Soil samples were collected at fixed intervals along each transect, for a total of five soil samples—one sample per zone—per wetland.

All 45 wetlands were sampled between June and August 2021. Soil cores were obtained using a soil core sampler equipped with plastic liner (7.6 cm *×* 15 cm; AMS 404.74, American Falls, ID, USA). Cores and liners were extruded from the soil core sampler, capped at both ends, then sealed in plastic bags, transported back to the lab on ice, and stored (4$^\circ$C) until further processing.

Soil samples were analyzed for three parameters: bulk density, carbon content and aggregate content. In the lab, soil cores were weighed, and measured accurately for diameter and length. Subsamples of the field-moist soil core were used for determination of soil bulk density. Remaining soil was air-dried, then subsampled for soil carbon content and aggregation.

Bulk density was determined by the core method (Blake 1965). For each soil sample, a *∼* 20 g subsample of the field-moist soil was dried (105 $^\circ$C) for 1–2 days. Bulk density ($\rho _b$) was then estimated as the quotient of the sample dry mass divided by the field-moist volume.

This bulk density estimator is also referred to as the uncorrected or total bulk density (total soil mass / volume; $\rho _{b,T}$). Bulk density can also be estimated with correction for gravel and coarse organic matter (fine-fraction mass / fine-fraction volume; $\rho _{b,F}$) or as a hybrid of the two (fine-fraction mass / total soil volume; $\rho _{b,H}$) (reviewed in Throop et al. 2012). The fine-fraction bulk density is expected to be a better indicator of overall soil condition (see Supplementary Section S5 and Supplementary Figs. S5–S7), therefore we did also estimate $\rho _{b,F}$ (as well as $\rho _{b,H}$). The total bulk density estimates, however, correlated strongly with the fine-fraction estimates (*r = 0.99*). Further, $\rho _{b,T}$ and $\rho _{b,F}$ performed similarly in preliminary statistical analyses. Because $\rho _{b,T}$ is easier to estimate (i.e. does not require additional processing), we opted to use $\rho _{b,T}$ in lieu of $\rho _{b,F}$ as our estimate for soil bulk density (henceforth $\rho _b$).

Soil carbon content (*SoilC*) was determined on a combustion-based elemental analyzer at an external lab. Subsamples (*∼* 1 g air-dried soil) were ground to pass through a 0.212 mm sieve (No. 10), packed in tin capsules, and shipped to the Washington State University Stable Isotope Core Laboratory (Pullman, WA, USA) for analysis. The precision of the elemental analyzer for *SoilC* was 0.8 % (range of 0.2 % to 2.1 %).

Water-stable aggregates (WSA) were quantified using an Eijkelkamp Wet-Sieving Apparatus (Wilmington, NC, USA). In brief, soil samples (*<8* mm diameter) were first dry-sieved at 2 mm, 1 mm, 0.5 mm and 0.25 mm. Approximately 4 g from each size fraction were then capillary-wetted and wet-sieved using a sieve with the corresponding lower bound (i.e. 2, 1, 0.5 or 0.25 mm). This procedure was modified from Kemper and Rosenau (1986) and Nimmo and Perkins (2002); see also Saygin et al. (2012), specifically method M$_{II}$.

The proportion of water-stable aggregates for each size fraction was calculated as

1 $$\begin{aligned} WSA_i = \frac{m_{a,i}}{(m_{a,i}+m_{u,i})} \end{aligned}$$

where *i* indicates the size fraction (2–8 mm, 1–2 mm, 0.5–1 mm or 0.25–0.5 mm); $m_{a,i}$ is the mass of water-stable aggregates (corrected for dispersant mass) for size fraction *i*; and $m_{u,i}$ is the mass of unstable aggregates. Note that in this calculation, any coarse particles (e.g. sand) in the initial 4 g of mass have been removed (i.e. $m_{a,i}$ + $m_{u,i}$ + coarse particles = 4 g; Six et al. 2002).

From the calculations of $WSA_i$, the mean weight diameter (*MWDc*; the “c” indicates this *MWD* is corrected for coarse-particles) was calculated as

2 $$\begin{aligned} MWDc = \frac{\sum {WSA_i \times \bar{d}_i}}{4} \end{aligned}$$

where $WSA_i$ is the proportion of water-stable aggregates (coarse-particle corrected) for size fraction *i*; and $\bar{d}_i$ is the mean diameter of size fraction *i* (e.g. for the 2–8 mm size fraction, $\bar{d}_i = (2+8)/2 = 5$ mm). The denominator of 4 reflects the equally-weighted average of 4 size fractions (i.e. each size-fraction analysis began with an initial 4 g of mass). The *MWDc* estimates have units of mm. More aggregated soils have higher *MWDc* values.

For all soil analyses, duplicates were analyzed every 20 samples. This resulted in the analysis of 12 duplicates out of 225 total samples for a frequency of 5 %. Analytical precision for the three soil parameters ($\rho _b$, *SoilC* and *MWDc*) was estimated using the root mean square (RMS) of the scaled relative differences between duplicate analyses (sometimes referred to as the mean coefficient of variation; Hyslop and White 2009). The estimates for RMS precision were 6.2 % for $\rho _b$, 1.6 % for *SoilC*, and 13 % for *MWDc*.

### Statistical Analyses

Figure generation, data manipulation and all statistical analyses were performed in R v. 4.0.5 (R Core Team 2021). Multivariate analyses (e.g. principal component analysis and permutational multivariate analysis) required the R packages *vegan*, *cluster* and *MVN*. Analysis of spatial data required the R packages *rgdal*, *maptools*, *rgeos*, *sp*, *remotes* and *polylabelr*. Metric evaluation required the R package *caret*.

#### Weighted Averages

For most statistical analyses, wetland site was the unit of replication. We therefore calculated weighted averages for $\rho _b$, *SoilC* and *MWDc*. Sample weights ($w_i$) per wetland were based on the areal proportion of the zone from which the soil sample was collected (see also Supplementary Section S3 and Supplementary Fig. S1), i.e. $w_i = a_i/A$, where $a_i$ is the area for zone *i* and *A* is the total area (i.e. $\sum a_i$). Weighted averages were then calculated as $\sum {w_i x_i}$, where $x_i$ is the sample value for zone *i* for the parameter of interest.

#### Sample Distribution and Normality

Prior to statistical analysis, we assessed the data for normality. Univariate normality for $\rho _b$, *SoilC* and *MWDc* was assessed by visual inspection of histograms and the Shapiro-Wilk test for normality. $\rho _b$ appeared just slightly right skewed, while *SoilC* was heavily right skewed and $MWD_c$ was heavily left skewed. Untransformed data were not normally distributed based on the Shapiro-Wilk test: $\rho _b$, *W=0.94*, *p=0.024*; *SoilC*, *W=0.87*, *p<0.001*; *MWDc*, *W=0.87*, *p<0.001*. The following transformations achieved normality based on the Shapiro-Wilk test (all *p> 0.05*): $\rho _b$, square-root transformation; *SoilC*, natural log transformation; *MWDc*, reflection then square-root transformation (i.e. $-1 \times \sqrt{\max MWDc - MWDc}$; note that, multiplication by -1 was applied to preserve the order).

Multivariate normality for $\rho _b$, *SoilC* and *MWDc* was assessed by visual inspection of chi-squared Q-Q plots and Mardia’s and Royston’s tests for normality (Oppong and Agbedra 2016). Collectively, the untransformed data were not normally distributed: e.g. Mardia skewness 38.7, *p < 0.001*; Royston *H = 25.6*, *p < 0.001*. Transformation of all three metrics, however, resulted in multivariate normality: e.g. Mardia skewness 12.1, *p = 0.278*; Royston *H = 3.3*, *p = 0.178*. We also tested a dataset with *SoilC* and *MWDc* transformed, but $\rho _b$ untransformed. This dataset also passed the tests for multivariate normality: e.g. Mardia skewness 13.7, *p = 0.190*; Royston *H = 4.5*, *p = 0.100*.

Based on the above data explorations, for all subsequent statistical analyses, we applied the natural log transformation to *SoilC* and reflection then square-root transformation to *MWDc*. We left $\rho _b$ untransformed because that did not affect multivariate normality and because the slight univariate skewness reflected the slightly greater proportion of higher quality wetlands in the sample set. Additionally, it was preferred that the proposed soil-based ORAM metric be based on the untransformed bulk density values.

#### Soil Health and Current ORAM

Principal component analysis (PCA; “rda” function in R package *vegan*) was used to evaluate overall soil health based on $\rho _b$, *SoilC* and *MWDc*. Site scores along the first principal component (PCA1) were additionally used as an index for overall soil health.

The relationship between overall soil health and current ORAM category was tested by permutational multivariate analysis of variance (PERMANOVA; “adonis” function in R package *vegan*). The relationship between overall soil health and current ORAM score was tested by simple linear regression using PCA1 as the response variable.

#### Soil Bulk Density Metric Development

Some of the more quantitative metrics in the current ORAM assigned points based on bin assignment (e.g. Metric 1 for wetland size; Mack 2001a). In a similar manner, the general approach was to generate a set of ordered bins (e.g. 4) based on $\rho _b$, with each bin resulting in a different point assignment. We ultimately evaluated five potential 4-bin metrics based on $\rho _b$ (Methods 1–5). Each 4-bin metric consisted of three bin cut-offs.

We began with a simple 4-bin metric with bin cut-offs assigned according to $(\max - \min )/4$ (Barbour et al. 1999; Mack et al. 2000). Some references recommend capping at the 95th and 5th quartiles to eliminate outliers; however, the $\rho _b$ values being used were weighted-averages so some outlying values were already down-weighted. Nonetheless, we considered thresholds based on the full $(\max - \min )$ range (aka. Method 1) and the $(0.95\max - 0.05\min )$ range (Method 2).

Other potential break points were identified quantitatively with *k*-medoids clustering of $\rho _b$ and PCA1 (function “pam” in R package *cluster*, with *k=4*). $\rho _b$ and PCA1 were analyzed separately. Medoid clusters were ordered by increasing PCA1 (or equivalently decreasing $\rho _b$). Bin cut-offs were then estimated as the middle values separating the ordered clusters. The 4-bin metric based on the $\rho _b$ clusters formed the basis of our Method 3 metric. For the Method 4 metric, bin cut-offs were based on the PCA1 clusters after expression in terms of $\rho _b$. This conversion used coefficients from the simple linear regression of $\text {PCA1} \sim \rho _b$; the conversion formula was then $\rho _{b,cutoff} = (\text {PCA1}_{cutoff} - b)/m$, where *b* is the regression intercept, *m* is the regression slope, and $\rho _{b,cutoff}$ and $\text {PCA1}_{cutoff}$ are bin cut-offs expressed as $\rho _b$ and PCA1.

For Method 5, potential bin cut-offs were optimized using linear regression. Initial bin cut-offs were averaged from the two best metrics out of Methods 1–4 (based on classification accuracy). Bin cut-offs were then sequentially searched across a small range to determine the value that maximized the coefficient of determination for $\text {PCA1} \sim \rho _b$ -based bin assignment.

#### Metric Evaluation

The five potential $\rho _b$-based metrics (Methods 1–5) were evaluated by repeated stratified k-fold cross validation, using 10 folds and 10 repeats (*caret* package in R). Stratification was by ORAM category. Metrics were evaluated on (1) classification accuracy and (2) simple linear regression. For classification accuracy, wetland sites were assigned to a $\rho _b$-bin based on the weighted-average $\rho _b$ and the bin cut-offs for the method. These were considered to be the “predicted” soil health classes. “Actual” or true soil health classes were considered to be the cluster assignment based on *k*-medoids clustering of PCA1 scores (i.e. the initial cluster assignments; not the Method 4 bin assignments following conversion of PCA1-based cut-offs to $\rho _b$ cut-offs). Classification accuracy was then the percentage of wetland sites correctly classified into its soil health bin (“actual”) according to $\rho _b$ (“predicted”).

Simple linear regression provided evaluation independent of constructed soil health bins. For this evaluation, $\rho _b$-bin assignments were assessed based on how well they predicted PCA1 scores. We used the adjusted coefficient of determination as the assessment measure because it adjusts for sample size and number of parameters. Both sample size and number of parameters could vary across the validation folds. Sample size in the validation folds averaged 4.5 and ranged from 3 to 6. The number of parameters (i.e. bins) could range from 1 to 4 and varied by validation sample and method being evaluated.

#### Metric Incorporation into ORAM

The current ORAM (v5.0) has six metrics, with a combined fourteen submetrics (Mack 2001a). Metric 3 (with five submetrics) is worth a maximum of 30 points and scores attributes of hydrology; Metric 6 (with four submetrics) is worth a maximum of 20 points and scores attributes of vegetation. Hydrology and vegetation are two of the three requisite wetland components, with soil being the third (Environmental Laboratory 1987; Federal Interagency Committee for Wetland Delineation 1989). If a new soil-based metric were added, at minimum it should receive equal weighting with vegetation.

We proposed a new “soil metric” or Metric 7, comprised of two submetrics. One submetric (7b) would be the current ORAM Submetric 4a which rates substrate disturbance and is worth a maximum of 4 points. The other submetric (7a) would be based on our 4-bin $\rho _b$ metric and worth initially a maximum of 16 points (so that Metric 7 is worth a total of 20 points; i.e. equal with the vegetation Metric 6). Addition of the new Metric 7 at a 20-point maximum to the current ORAM required rescaling all point assignments to preserve an ORAM score maximum total of 100. The resulting revised ORAM with seven metrics (v6.0) had point maximums of 12 for Submetric 7a, 4 for Submetric 7b, 26 for Metric 3 (hydrology), 17 for Metric 6 (vegetation), and 41 for all remaining. (Note that, with this rescaling, Metric 7 is slightly below Metric 6 in terms of maximum point contribution.) Specifically for Submetric 7a, point assignments for the four bins ordered highest soil health/lowest $\rho _b$ to lowest soil health/highest $\rho _b$ were 12, 8, 4 and 1.

## Results and Discussion

### Assessment of Soil Health

**Fig. 2:**
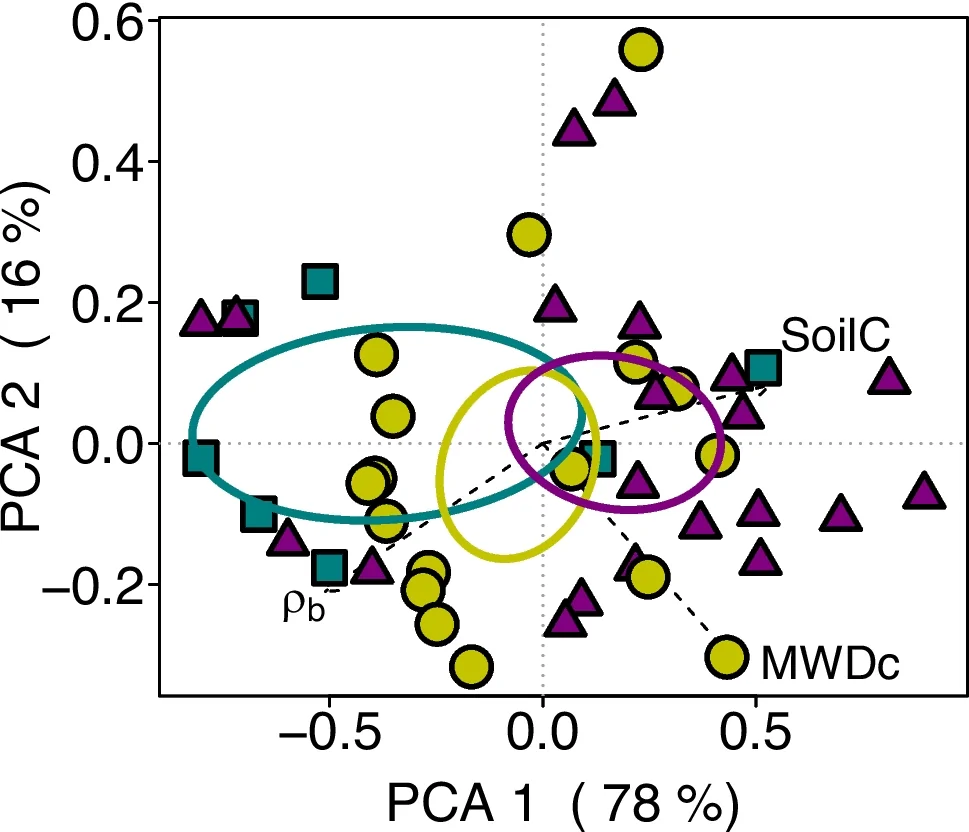
Principal component analysis (PCA) was applied to the three measurements of soil health. The primary axis explained 78 % of the variation in the data and correlated strongly with all three soil properties. Wetlands are shaped and colored by ORAM v5.0 category: green squares, Category 1; yellow circles, Category 2; purple triangles, Category 3. The three ellipses indicate 95 % confidence intervals around the centroids of the three ORAM categories

To assess overall soil health we performed a PCA on the three measurements of soil health (Fig. 2). The primary axis (PCA1) explained 78 % of the variation in the data and correlated strongly to all three soil properties: $\rho _b$, *r=-0.89*; *SoilC*, *r=0.93*; *MWDc*, *r=0.82*. Healthier soils are expected to have low $\rho _b$, high *SoilC* and high *MWDc*. The same properties were only moderately to weakly correlated to the secondary axis (PCA2), which explained only 16 % of the variation in the data: $\rho _b$, *r=-0.37*; *SoilC*, *r=0.15*; *MWDc*, *r=-0.56*. Because of the weaker correlations and lower variance explained for PCA2, we focused only on PCA1. Henceforth, PCA1 was considered the index for soil health, with higher PCA1 scores corresponding to healthier soils.

### Correlation Between ORAM v5.0 and Soil Health

The relationship between overall soil health ($\rho _b$, *SoilC*, *MWDc*) and ORAM category was statistically significant ($R^2 = 0.14$, $F_{2,42} = 3.52$, *p=0.022*); all post-hoc pairwise comparisons, however, were statistically insignificant at *α = 0.05* (FDR adjusted). The strongest separation was between Category 3 and Category 1 wetlands (FDR-adjusted *p=0.051*). The overall weak separation between ORAM categories was apparent by the overlap in 95 % confidence ellipses between Category 3 and Category 2, and between Category 1 and Category 2 (Fig. 2). Visual inspection also revealed little discernible pattern in ORAM quality in the two dimensional ordination.

**Fig. 3:**
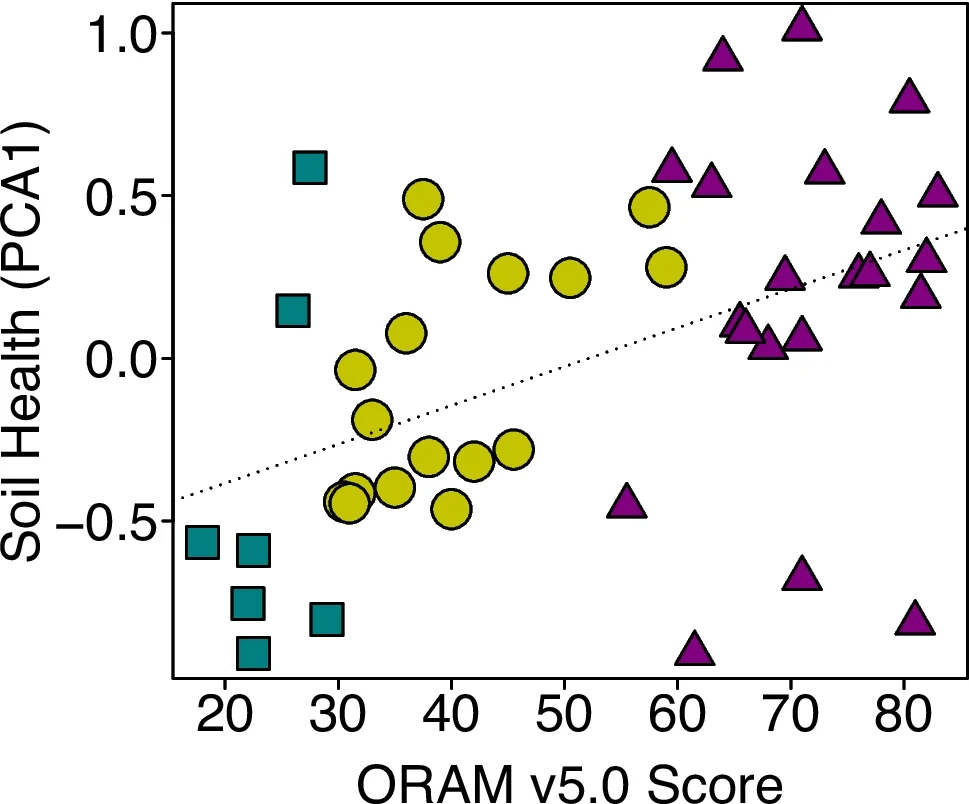
Soil health (PCA1) correlated with ORAM v5.0 score; despite the statistical significance, however, the coefficient of determination was low ($R^2=0.23$, $F_{1,43} = 12.7$, *p < 0.001*). Furthermore, there were notable outliers: two Category 1 wetlands had high soil health scores; four Category 3 wetlands had low soil health scores. The intercept and slope for the best fit regression line (dotted line) were -0.62 and 0.012. Wetlands are shaped and colored by ORAM v5.0 category: green squares, Category 1; yellow circles, Category 2; purple triangles, Category 3

Wetland scores for PCA1 (i.e. the soil health index score) were compared to current ORAM scores by simple linear regression (Fig. 3). While the relationship was statistically significant, the variation in soil health explained by ORAM v5.0 score was low ($R^2 = 0.23$, $F_{1,43} = 12.7$, *p < 0.001*). This low coefficient of determination contrasts with previous assessments of the ORAM in comparison to disturbance, vegetation quality, and other biological indicators. These comparisons resulted in much higher coefficients of determination ranging from $R^2=0.66$ to $R^2=0.92$, supporting the reliability of the ORAM as an indicator for these qualities (Fennessy et al. 1998; Mack et al. 2000). In contrast, the current ORAM is a less reliable indicator of soil health (based on the coefficient of determination). There were also notable outliers: e.g. two Category 1 wetlands (with low ORAM v5.0 scores) had high soil health scores; four Category 3 wetlands (with high ORAM v5.0 scores) had low soil health scores. These evaluations suggest that incorporating a soil-based metric into the current ORAM could improve its representation of soil health (i.e. a revised ORAM would be a better soil health indicator) and thus overall wetland condition.

### Suitability of Bulk Density as a Proxy for Soil Health

We proposed $\rho _b$ as the basis for a metric to include in the ORAM (v5.0) to better reflect soil health and overall wetland condition. Although *SoilC* was the best indicator of soil health (*r = 0.93*; “Assessment of Soil Health”), $\rho _b$ was also a strong indicator (*r = -0.89*). Additionally, while both $\rho _b$ and *SoilC* correlate well with many soil properties, $\rho _b$ is relatively easier/cost-efficient to obtain. $\rho _b$ also has been shown to respond more quickly to change than does *SoilC* (Hossler and Bouchard 2010; Or et al. 2021).

#### Correlation with Other Soil Properties

Several previous studies have demonstrated that $\rho _b$ correlates well with many soil properties and indicators of soil function (Rokosch et al. 2009; Hossler et al. 2011; Fennessy and Wardrop 2016; Onufrak et al. 2020). This correlation was also confirmed for the sample set of 45 wetlands. Comparisons of $\rho _b$ directly with *SoilC* and *MWDc* revealed strong to moderate correlations on a per wetland basis (i.e. using weighted mean values, *n = 45*): *r=-0.80* and *r=-0.55*. On a per soil sample basis (*n = 225*), the correlations of $\rho _b$ to *SoilC* and *MWDc* were *r=-0.76* and *r=-0.51*. (*SoilC* had a slightly stronger correlation with *MWDc* than did $\rho _b$: *r=0.66*, per wetland basis; *r=0.64*, per soil sample basis.)

#### Ease of Measurement and Cost Efficiency

In terms of cost efficiency of $$\rho _b$$, our soil core sampler plus slide hammer cost $700. The other instrumentation required for measurement of $$\rho _b$$ was a basic balance and drying oven—typical equipment for most labs. For comparison, determination of *SoilC* required a semi micro balance (0.001 mg precision) for weighing out the soil samples. Semi micro balances typically cost over *$15,000*. The samples were analyzed externally at a cost of $5.5 per sample. Determination of aggregate content required a wet-sieving apparatus. The cost of this apparatus plus the necessary sieves was $6,500.

In terms of ease of measurement, once the soil core was collected, the determination of $\rho _b$ required measurement of the core length for volume determination; weighing the field-most soil core; then drying and weighing a subsample for determination of dry mass. For comparison, determination of *SoilC* required air-drying the soil then grinding a subsample to pass a 0.212 mm sieve. A very small sample of the fine soil (6–10 mg) was then packed in a *5× 9* mm tin capsule and shipped to the external lab. Determination of *MWDc* was the most laborious and required sieving the air-dried soil through a series of sieves. Fractions collected on each “dry sieve” were then placed on corresponding “wet sieves.” Each wet sieve fraction then went through a sequence of capillary wetting; sieving in distilled water for an allocated time; collection, drying and weighing of “unstable” aggregates; sieving the remaining aggregates in a dispersant solution (e.g. 0.2 % hexametaphosphate); collection, drying and weighing of the “stable” aggregates; then collection, drying and weighing of any remaining coarse particles.

### The Soil Bulk Density Metric

We targeted a four-bin bulk density metric. Each bin would be assigned a point score. The bin representing the lowest $\rho _b$ (i.e. highest soil quality) would be scored highest, and the bin representing the highest $\rho _b$ (i.e. lowest soil quality) would be scored lowest.

#### Identification of Break Points

**Fig. 4:**
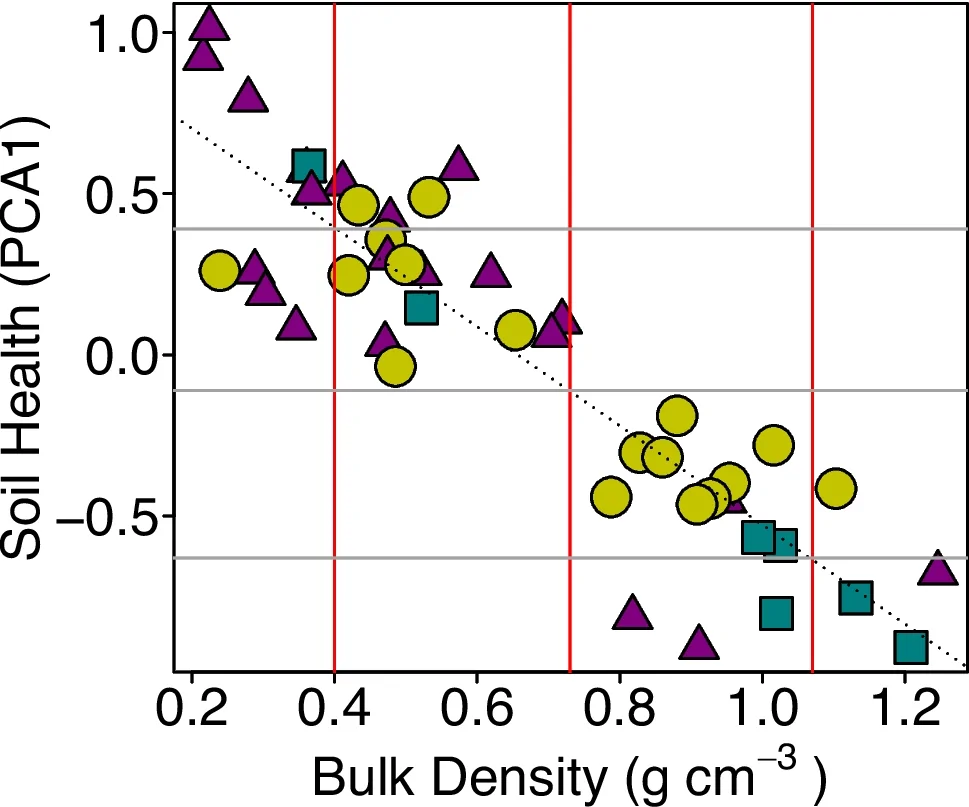
Wetlands are plotted according to soil health score (PCA1; y-axis) and $\rho _b$ (x-axis). The dotted black line shows the best fit line by linear regression ($R^2 = 0.79$, $F_{1,43} = 166.4$, *p < 0.001*). The vertical red lines indicate the recommended $\rho _b$ bin cut-offs based on Method 4: 0.40, 0.73 and 1.07 g cm$^{-3}$. The horizontal gray lines indicate the corresponding soil health cut-offs 0.39, -0.11, -0.63. Wetlands are shaped and colored by ORAM v5.0 category: green squares, Category 1; yellow circles, Category 2; purple triangles, Category 3

Preliminary bin assignments based on the full $(\max - \min )$ range (Method 1) were *<0.47*, 0.47–0.73, 0.73–0.99 and *>0.99* g cm$^{-3}$. These bin assignments were similar to those based on the $(0.95\max - 0.05\min )$ range (Method 2): *<0.47*, 0.47–0.69, 0.69–0.91, and *>0.91* g cm$^{-3}$. Bins based on the $(\max - \min )$ range, however, provided better separation. For example, the plot of overall soil health (PCA1) against $\rho _b$ indicated a clear break in soil health scores around -0.11 (Fig. 4), which corresponded to *∼ 0.73* g cm$^{-3}$ $\rho _b$. Thus at minimum, wetlands with $\rho _b < 0.73$ g cm$^{-3}$ should be scored higher (healthier) and wetlands with $\rho _b> 0.73$ g cm$^{-3}$ should be scored lower (less healthy).

Clustering of $\rho _b$ values (Method 3) suggested bins *<0.39*, 0.39–0.68, 0.68–0.92, and *>0.92* g cm$^{-3}$. Clustering of PCA1 scores suggested PCA1 cut-offs of -0.63, -0.11 and 0.39. Conversion of these cut-offs to $\rho _b$ using the coefficients from the regression of PCA1 on $\rho _b$ (e.g. Figure 3; Method 4) resulted in bin assignments of *<0.40*, 0.40–0.73, 0.73–1.07, and *>1.07* g cm$^{-3}$. Method 5 optimized bin cut-offs averaged from Methods 1 and 4 (current two best methods based on classification accuracy). This optimization resulted in suggested bins of *<0.42*, 0.42–0.75, 0.75–1.11, and *>1.11* g cm$^{-3}$.

Classification accuracy to PCA1 clusters and $R^2_{adj}$ to PCA1 scores for the entire dataset were highest for Method 5 (0.76, 0.81), followed by Method 1 (0.71, 0.80) and Method 4 (0.71, 0.79) (Table 2). When evaluated by repeated k-fold cross validation, however, Method 4 was the most robust across the sample iterations, with accuracy and $R^2_{adj}$ scores of 0.67 and 0.72. Methods 1 and 5 had slightly lower performance, with accuracy and $R^2_{adj}$ scores of 0.65 and 0.71 (Table 2). Because of the better performance by Method 4 in the cross validation assessment, we selected these $\rho _b$ bin cut-offs as our basis for a $\rho _b$ soil metric (Fig. 4).

**Table 2:** Evaluation of $\rho _b$ bin assignments based on Methods 1–5. Methods were assessed by (1) classification accuracy (ACC) to PCA1 clusters and (2) adjusted $R^2$ to PCA1 scores, using the full dataset and repeated k-fold cross validation (k-fold CV)

	full dataset		k-fold CV	
Method	ACC	$R^2_{adj}$	ACC	$R^2_{adj}$
1 (max–min)	0.71	0.80	0.65	0.71
2 (0.95 max–0.05 min)	0.58	0.75	0.61	0.65
3 ($\rho _b$ clustering)	0.56	0.75	0.63	0.68
4 (PCA1 clustering)	0.71	0.79	0.67	0.72
5 (optimization)	0.76	0.81	0.65	0.71

#### Evaluation of Revised ORAM (v6.0)

The $\rho _b$ soil metric was then incorporated into the current ORAM as part of a new “soil metric” or Metric 7. The resulting revised ORAM v6.0 was comprised of seven metrics: the new 16 point Metric 7 (soil)—which in turn was comprised of a 12-point $\rho _b$ metric (Submetric 7a) and a 4-point soil disturbance metric (Submetric 7b; formerly Submetric 4a in ORAM v5.0)—and ORAM v5.0 Metrics 1–6, reweighted to maintain a maximum ORAM score of 100 points (with exception of Submetric 4a which was incorporated into the new Metric 7; see also “Metric Incorporation into ORAM” in the Methods).

Revised ORAM scores (i.e. v6.0) were highly correlated with scores based on ORAM v5.0 for the 45 wetlands in this study (*r=0.99*). Under ORAM v6.0, five wetlands were recategorized: two Category 1 wetlands were upgraded to Category 2; one Category 2 wetland was upgraded to Category 3; one Category 2 wetland was downgraded to Category 1; and one Category 3 wetland was downgraded to Category 2. These recategorized wetlands included three of the previously identified outliers: two with low ORAM scores but high soil health; and one with a high ORAM score but low soil health (Fig. 3). Correlations to soil health and $\rho _b$ improved with incorporation of the soil-based metric: the correlation with soil health (PCA1) was *r=0.48* for v5.0 and improved to *r=0.58* for v6.0; the correlation with $\rho _b$ was *r=-0.53* for v5.0 and improved to *r=-0.64* for v6.0. (Note, evaluation of Submetric 7a using soil health [PCA1] scores rather than $\rho _b$ as proposed resulted in only marginal improvement in the correlation strength between v6.0 and soil health [*r=0.61*] and there was no change in the correlation strength between v6.0 and $\rho _b$ [*r=-0.64*].)

**Fig. 5:**
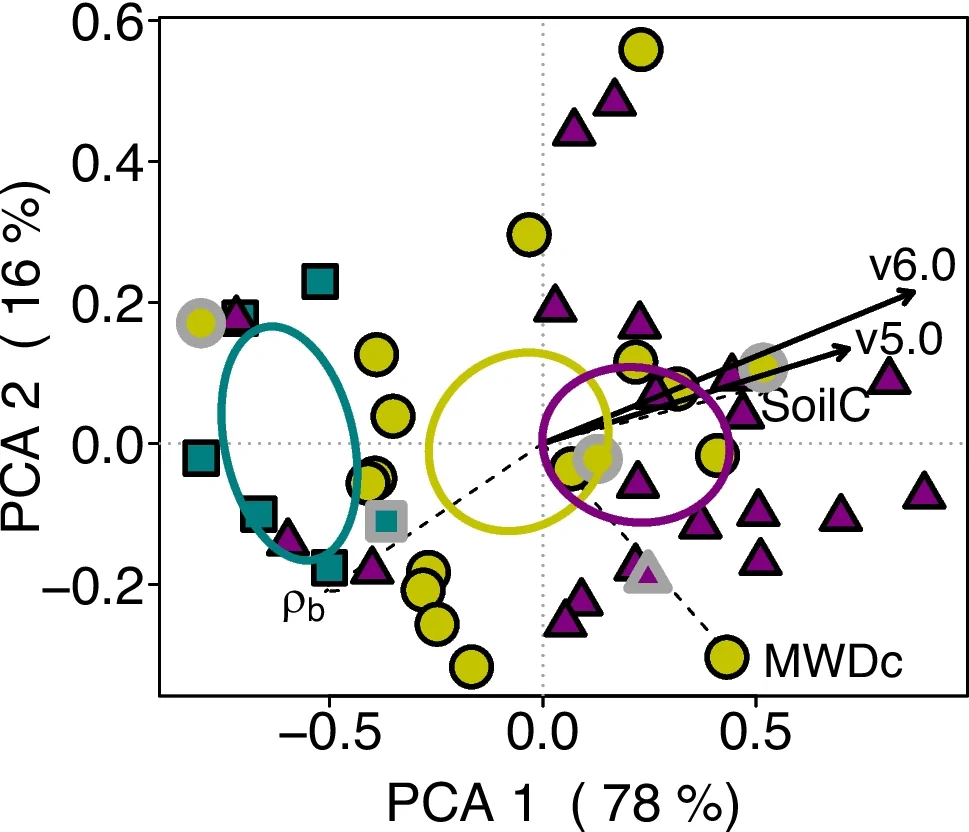
Wetland categorization and ORAM score under the revised v6.0 were compared to the PCA ordination of soil health (compare to Fig. 2). In this ordination, wetlands are shaped and colored by ORAM category (v6.0): green squares, Category 1; yellow circles, Category 2; purple triangles, Category 3; the five wetlands recategorized under ORAM v6.0 are outlined in gray. The three ellipses indicate 95 % confidence intervals around the centroids of the three ORAM categories (v6.0). Projections of ORAM v6.0 and ORAM v5.0 scores are also indicated with the solid black arrows. (Note, for readability, the arrows for v6.0 and v5.0 are not on the same scale as the soil property arrows)

Wetland category assignment under the revised ORAM (v6.0) was also compared to soil health by PERMANOVA. The overall relationship was statistically significant ($R^2 = 0.29$, $F_{2,42} = 8.41$, *p<0.001*), as well as two of the post-hoc pairwise comparisons (i.e. FDR-adjusted *p≤ 0.05*) (Fig. 5). Only categories 2 and 3 had poor separation (FDR-adjusted *p=0.058*). Recall, the PERMANOVA between the current ORAM v5.0 and soil health found no statistically significant pairwise differences (e.g. Fig. 2). ORAM scores under v5.0 and v6.0 were also fit to the soil health PCA ordination (Fig. 5). The incorporation of the conservative 12-point $\rho _b$-based metric improved the correlation of ORAM score to overall soil health (i.e. based on ORAM score projections onto the soil health PCA ordination; v6.0 *r=0.60*, v5.0 *r= 0.49*).

It should be noted, that the current 12-point Submetric 7a is conservative: e.g. under ORAM v6.0, maximum point contributions are 26 % for hydrology (Metric 3), 17 % for vegetation (Metric 6) and 16 % for soil (Metric 7). Obviously, higher weighting of the bulk-density based submetric (7a) would improve the representation of soil health. Increasing the point assignment for Submetric 7a to 20 to reach full equivalence with Metric 3 (with both Metric 7 and Metric 3 contributing 24 % maximum), for example, increases the correlation of ORAM v6.0 with PCA1 and $\rho _b$ to *r=0.64* and *r=-0.71* and only decreases the correlation between ORAM v6.0 and ORAM v5.0 to *r=0.97* (Supplementary Fig. S8a,b and Supplementary Table S1; see also Supplementary Section S6). How metrics are weighted is an important consideration for rapid assessment methods like the ORAM, where the final result is one weight-averaged score (Fennessy et al. 2007). The purpose of a soil-based metric is to improve representation of the soil condition and valuation of soil-based functions, but inadvertently this may down-weight other aspects of condition and function (e.g. hydrology and hydrologic functions). Notably, wetland function and capacity will vary by wetland type (e.g. HGM class; Smith et al. 2013). Fennessy et al. (2007) discuss options for handling this inherent variability, such as having different sets of metrics for different wetland types (e.g. State of Washington 1993). Another option—at least for the ORAM—would be to use $\rho _b$ to justify recategorization and award extra points as part of a “value-added-metric” (Metric 5 in the OrAM; Fennessy et al. 2007; Mack 2001a; see also “Alternative ORAM Revisions to Encompass Soil Health” below).

### Alternative ORAM Revisions to Encompass Soil Health

In lieu of directly incorporating a soil-based metric into the current ORAM, two alternative revisions are (1) reweight the current six ORAM metrics to better reflect soil health; and (2) use $\rho _b$ to justify recategorization and award extra points to Metric 5. Alternative (1) is described and assessed in Supplementary Section S7. In brief, the reweighting option offered only modest improvement over the proposed ORAM v6.0, and was achieved with complete down-weighting of Metrics 2, 3 (hydrology) and 5. Clearly this option would not meet the objective of better representation across all three primary wetland components: hydrology, vegetation and soil.

Alternative (2) is discussed in Supplementary Section S8. Under this alternative, wetlands with a mean soil $\rho _b$ above a certain threshold could be assigned to Category 3 or Category 2 status in spite of a potentially lower ORAM score. We recommend that wetlands with mean bulk densities *<0.40* g cm$^{-3}$ be delegated Category 3, and wetlands with mean bulk densities *≤ 0.73* g cm$^{-3}$, be delegated Category 2 or higher (Supplementary Fig. S9a). This option can also be quantified using Metric 5 of the current ORAM (Supplementary Fig. S9b). Metric 5 specifically adds or deducts points based on the narrative questions. The advantage of this alternative is that it would preserve the current ORAM v5.0 scoring system, but provide a formal mechanism to prioritize wetlands with well-developed high-quality soils. Wetlands would only be upgraded because of good soil conditions, but not downgraded because of poor soil conditions.

### Additional Considerations

#### Covariates

Soil condition is expected to be sensitive to HGM and vegetation class (e.g. topography and organisms are two of the main soil forming factors; Jenny 1941; see also Noble and Berkowitz 2016; Daugherty et al. 2019), as well as soil texture (Gupta and Larson 1979; Arshad and Coen 1992; Hassink 1997; Bünemann et al. 2018; Maurya et al. 2020; Matus 2021). Lane et al. (2025), for example, compared soil carbon content across wetlands of different HGM class and observed statistically higher *SoilC* (0–10 cm) in slope and depression wetlands compared to riverine wetlands. Numerous studies in the broader literature have linked finer soil texture (i.e. higher clay content) to both lower $\rho _b$ and higher *SoilC* (e.g. Adams 1973; Hassink 1997; Ruehlmann and Körschens 2009; Hollis et al. 2012; Matus 2021).

**Fig. 6:**
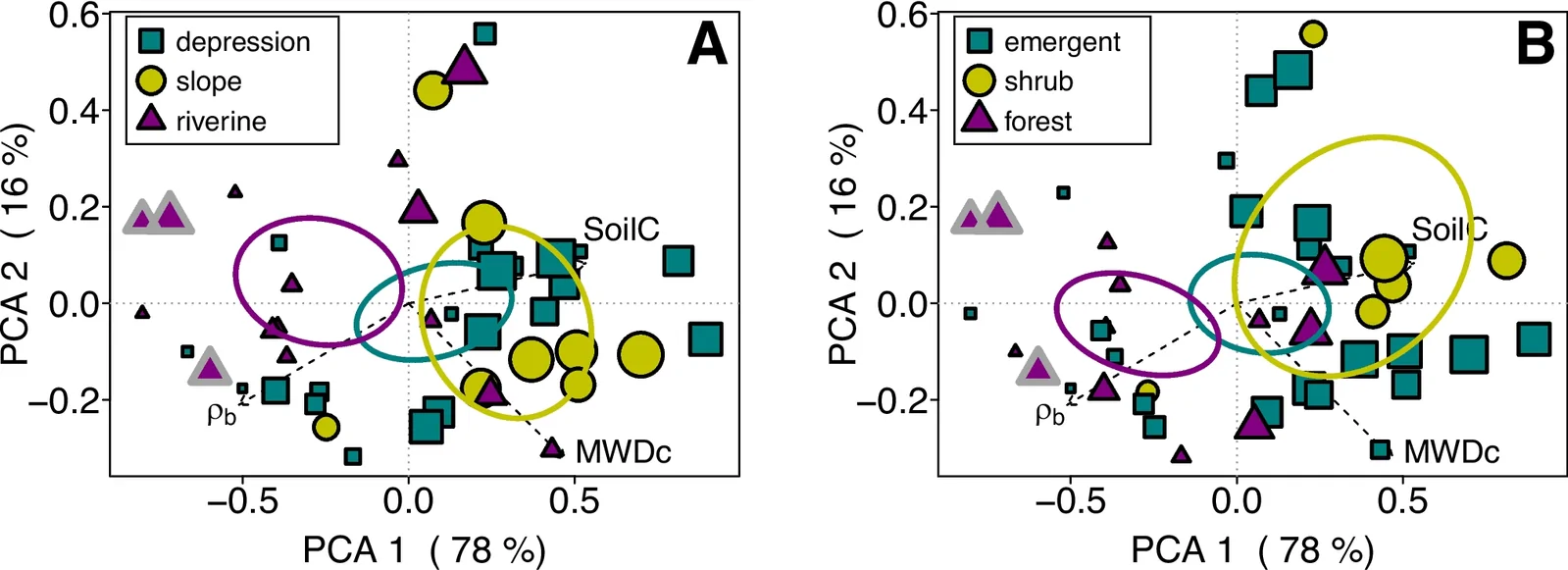
Principal component analysis (PCA) was applied to the three measurements of soil health. Overall soil health correlated to both hydrogeomorphic class (HGM) and dominant vegetation. In (**a**), points are shaped and colored by HGM class and sized by ORAM v5.0 score (e.g. larger size = higher score). The ellipses indicate the 95 % confidence intervals around the centroids of the three HGM classes. In (**b**), points are shaped and colored by dominant vegetation and sized by ORAM v5.0 score. The ellipses indicate the 95 % confidence intervals around the centroids of the three vegetation classes. The three outlier wetlands are outlined in gray

In our study, overall soil health had a statistically significant relationship with both HGM class ($R^2 = 0.17$, $F_{2,42} = 4.37$, *p=0.011*; Fig. 6a) and dominant vegetation ($R^2 = 0.18$, $F_{2,42} = 4.70$, *p=0.007*; Fig. 6b). Specifically, wetland soil quality was lower (i.e. higher $\rho _b$, lower *SoilC* and *MWDc*) in riverine and forested wetlands. Unsurprisingly, there was a similar relationship between HGM and vegetation with PCA1: riverine wetlands scored statistically lower on the index (overall model fit by ANOVA: $R^2 = 0.21$, $F_{2,42} = 5.73$, *p = 0.006*); as did forested wetlands (overall model fit by ANOVA: $R^2 = 0.22$, $F_{2,42} = 6.09$, *p = 0.005*).

Some of these differences were due to a lack of low quality slope wetlands and shrub wetlands in our sample set. In spite of our efforts to achieve equal representation across HGM and vegetation classes, we ended up with no Category 1 slope wetlands or shrub wetlands. Pairwise differences for the relationships noted above were strongest between riverine and slope wetlands and between forest and shrub wetlands. Relationships of overall soil health with HGM and vegetation were therefore also assessed within ORAM v5.0 category. With this re-analysis, the only significant difference was that Category 3 forested wetlands had lower soil quality than Category 3 emergent and shrub wetlands. Similar results were obtained when assessed within the revised ORAM categories based on $\rho _b$ (i.e. ORAM v6.0).

The remaining differences in soil health were driven by three wetlands in particular: these three wetlands were both forested and riverine, Category 3 (under ORAM v5.0), and had low soil health scores. These three wetlands were among six wetlands identified as potential outliers warranting the potential inclusion of a soil-based metric in the current ORAM (e.g. Figure 3; see also Supplementary Fig. S10). When those three wetlands were excluded from the analysis, the relationship between soil health and HGM class was no longer significant (*p> 0.05*), and the relationship between soil health and vegetation class became only marginally significant (overall soil health: $R^2 = 0.12$, $F_{2,39} = 2.74$, *p = 0.047*; PCA1: $R^2 = 0.14$, $F_{2,39} = 3.29$, *p = 0.048*). Similar results were obtained for the relationships between $\rho _b$ and HGM and vegetation class (data not shown).

The effects of HGM and vegetation class and the three outliers were also evaluated by inclusion as interaction terms in comparisons of soil health to ORAM score (v5.0 and v6.0): i.e. *Soil Health* *∼* *ORAM* + *ORAM*:(*HGM* or *VEG*). There were significant interactions between ORAM score and both covariates when all 45 wetlands were assessed. When the three potential outliers were removed, however, these interactions became statistically insignificant. These results were consistent whether the relationship was assessed by PERMANOVA to overall soil health (Supplementary Table S2) or by simple linear regression to the soil health index (i.e. PCA1; Supplementary Table S3).

Overall, the assessment of soil health and the bulk density proxy for soil health appear robust across HGM and vegetation class for wetlands of west–central Ohio. The “outlying” nature of the three wetlands, however, brings an important point: additional soil forming factors should be considered when developing a soil-based metric for wetland assessment. HGM class, for example, is likely the factor impacting the soil properties of one of the three wetlands. This particular wetland was the only riverine wetland in our sample set that was directly adjacent to a river. Floodplain soils directly adjacent to rivers tend to be poorly developed because of the continual sedimentation and reworking from frequent flooding (e.g. Smith and Klimas 2002; Saint-Laurent et al. 2010). The frequent flooding, however, also can bring nutrients resulting in potentially high carbon content (e.g. Bai et al. 2020). Parent material may be driving the soil differences in the other two wetlands. These two wetlands were among only three in our sample set that were located in the unglaciated region of Ohio. In this region, the parent material tends to be sandstone and shale, whereas the parent material in the glaciated region is primarily limestone and glacial till. Soils in the unglaciated region are typically coarser in texture and contain less organic carbon (Tan et al. 2004; Ohio Department of Agriculture 2018).

Soil texture is another potential covariate—which is also dependent on soil forming factors such as parent material and topography (e.g. HMG) in particular (Ito et al. 2023). Soil texture was estimated using data available from the USDA Soil Survey Geographic (SSURGO) Database (Soil Survey Staff 2024) (see Supplementary Section S9). Note, that these estimates are approximate and will reflect the typical soil types of the locales within which the wetlands are located. The per-sample SSURGO-based soil properties (Supplementary Section S9) were first weighted-averaged by wetland (see “Weighted Averages”) before comparing to the per-wetland based estimates of soil health.

Overall soil health had statistically significant relationships with estimates of percent sand, percent clay, and geometric mean particle diameter ($d_g$) (Supplementary Fig. S11). Relationships with the soil health index (PCA1) were also statistically significant: sand, $R^2=0.14$, $F_{1,43}=7.0$, *p=0.011*; clay, $R^2=0.20$, $F_{1,43}=10.5$, *p=0.002*; and $d_g$ (log transformed), $R^2=0.21$, $F_{1,43}=11.6$, *p=0.001*. These relationships were independent of ORAM scores (both v5.0 and the revised v6.0; all $R^2 < 0.009$ and all *p> 0.05*) and thus were not indicative of any bias between soil health and ORAM score because of soil textural differences. Rather, these relationship were driven by the relationships of soil quality indicators *MWDc* and *SoilC* with $d_g$, percent sand, and percent clay (Supplementary Fig. S12). Notably, $\rho _b$—the soil quality indicator focused on in this study—demonstrated no statistically significant relationship with soil texture. This suggests that $\rho _b$ will be a robust indicator of soil health across a range of wetland soil types.

Although not explicitly explored here, region would be an additional potential covariate reflecting soil forming factors such as climate and parent material. Within Ohio, our sample sites were located primarily in the west–central region Ohio (Fig. 1). As mentioned earlier, most of our sites were located in the glaciated region, but three were located in the unglaciated (southeast) region (glaciation being a primary determinant of soil properties in Ohio; Tan et al. 2004; Ohio Department of Agriculture 2018). To help assess representation of our sample set for Ohio, we compared our measurements of $\rho _b$ and *SoilC* to measurements obtained by Gara and Schumacher (2015). The Gara and Schumacher (2015) study sampled 50 wetlands across Ohio, with concentration in the north and northeast regions of Ohio. The range of values were comparable: our study, $\rho _b$ 0.04–1.69 g cm$^{-3}$ and *SoilC* 1.24–42.7 g hg$^{-1}$; Gara and Schumacher (2015), $\rho _b$ 0.11–1.38 g cm$^{-3}$ and *SoilC* 1.19–51.4 g hg$^{-1}$. This suggests that our proposed $\rho _b$ thresholds for a soil-based metric should be applicable across Ohio, but should be verified.

Our values were also comparable to more global datasets consisting of wetland and upland samples: e.g. Ruehlmann and Körschens (2009), $\rho _b$ 0.03–2.00 g cm$^{-3}$ and *SoilC* 0.26–57.4 g hg$^{-1}$. Thus, our proposed $\rho _b$ thresholds for a soil-based metric may have broader applicability. However, this should be confirmed and adjusted as needed given documented regional differences in soil (e.g. Heuscher et al. 2005; Nahlik and Fennessy 2016). This would be consistent with the region-specific development of most wetland rapid assessment methods (e.g. Fennessy et al. 2007).

#### Organic Soil Material

Soil material is considered predominantly organic (i.e. organic soil material) if the fine soil fraction (i.e. *< 2.0* mm) is at least 12 % carbon (Soil Survey Staff 2022, Chapter 2). While many of the wetlands we sampled had soils with high concentrations of *SoilC*, only eleven met the threshold for “organic soil material” using per wetland weighted averages for *SoilC*. On a per soil sample basis, seven additional wetlands had at least one soil sample that met the maximum threshold (i.e. eighteen wetlands total; Supplementary Fig. S13).

The proposed bulk density metric placed seven of the eleven wetlands with organic soil material (per wetland basis) in the highest bin (i.e. $\rho _b < 0.40$ g cm$^{-3}$), and the remaining four (per wetland basis) in the second highest bin (i.e. $\rho _b$ 0.40–0.73 g cm$^{-3}$). On a per-soil basis, nine wetlands with at least one organic soil material sample were placed in the highest $\rho _b$ bin, and the other nine wetlands with at least one organic soil material sample were placed in the second highest $\rho _b$ bin (Supplementary Fig. S13). The bulk density metric is a proxy for overall soil health, for which organic/carbon content is a factor but not the sole determinant.

## Conclusion

Soil health was evaluated for 45 wetlands in west–central Ohio based on measurements of soil bulk density, carbon content and aggregation. For these wetlands, the current ORAM (v5.0) had a low correlation with soil health. Furthermore, there were notable outliers (e.g. Category 3 wetlands with low quality soils and Category 1 wetlands with high quality soils). This warrants consideration of soil in rapid assessments of wetland condition.

Bulk density is recommended to be used as a proxy for soil health in rapid wetland assessments. Soil bulk density has been shown to correlate well with many soil properties and indicators of soil function. In this study, soil bulk density correlated strongly with soil carbon content, aggregation and overall soil health. It is relatively easier and more cost efficient to obtain than other soil-based measurements, such as carbon content and aggregation.

For Ohio, a 12-point bulk density submetric was proposed to be included in a revised ORAM (v6.0). Addition of this conservative submetric improved the relationship of ORAM score and category to soil health. Attribution of higher points to the submetric resulted in further improvement.

Alternatively, soil bulk density could be used to justify recategorization of low-quality wetlands with high-quality soils. In the ORAM, for example, this could be accomplished by adding a narrative question that addresses soil bulk density and awarding points under Metric 5 for wetlands with good soil condition (based on soil bulk density). This mechanism would be a way to add value to wetlands with high quality soils and leave the current scoring system intact. This mechanism could also be used to account for different functional values in different types of wetlands (e.g. depression vs. riverine).

We recommend $\rho _b < 0.40$ g cm$^{-3}$ and $\rho _b < 0.73$ g cm$^{-3}$ as thresholds for highest quality and high quality soils. These thresholds may need to be adjusted by region to reflect differences in soil forming factors such as parent material, climate and topography.

Given the fundamental role of soil in many valued wetland functions and the long trajectory for soil development and recovery, it is highly important that soil be considered in rapid wetland assessments. These assessments are often the basis for prioritizing wetlands for protection. For the development of new rapid wetland assessments, states and tribes should consider adding a metric or submetric evaluating soil bulk density as a proxy for soil health. For states and tribes with rapid assessment methods in place, options for recategorization based on soil bulk density should be considered. These options would provide similar protections based on soil quality, with minimal modification of existing assessment methods.

## Supplementary Information

Below is the link to the electronic supplementary material.

Supplementary file 1 (pdf 616 KB)

## Data Availability

The datasets generated during and/or analyzed during the current study are available from the corresponding author on reasonable request.
