# Supplementary material for: A Soil Bulk Density Metric to Improve Rapid Assessment of Wetland Condition
Source: Wetlands (Wilmington). 2026 Jul 7;46(6):79. doi: 10.1007/s13157-026-02044-9 (PMC13341704; doi:10.1007/s13157-026-02044-9)
Supplement: Supplementary file 1 — (pdf 616 KB) [file 13157_2026_2044_MOESM1_ESM.pdf]

Supplementary Information

A soil bulk density metric to improve rapid assessment of wetland condition

Wetlands

Katie Hossler[1*], James Tyler Retherford[1], Mitchell Link[1]
[1]Department of Biological Sciences, Wright State University
*Corresponding author: katie.hossler@wright.edu

Supplementary Methods

Section S1 : Site selection

From a list of 299 wetland sites that had been previously scored using the ORAM (primarily by the Ohio EPA) and were in the target region of west–central Ohio, there were 256 potential sites that matched the six targeted HGM-vegetation subgroups (i.e. depression emergent, depression forest, depression shrub, riverine emergent, riverine forest, slope emergent). From the 256 potential sites, we selected 11 sites by purposive sampling (i.e. non-probability), as 'legacy' or 'special interest' sites. We then used the remaining 244 sites to generate our random sample of 39 sites, using a Generalized Random Tessellation Stratified (GRTS) survey design for a finite resource (Stevens and Olsen, 1999, 2004). The GRTS survey design helped ensure a geographically-balanced random sample, to increase precision and reduce bias (Stevens and Jensen, 2007; Lackey and Stein, 2013). The GRTS design has been implemented in several wetland surveys, including the most recent National Wetland Condition Assessment (USEPA, 2016; Gara and Schumacher, 2015; Olsen et al., 2019). In a manner similar to the NWCA, we employed a stratified, unequal probability design.

We utilized the *spsurvey* package in R v4.0.5. Our finite resource was the set of 256 potential sites, with each site represented as a point with latitude and longitude. Stratification was by ORAM category (i.e. ecological condition or wetland quality), with a target sample size of 13 per stratum. Within each stratum, the unequal selection probability categories were the six HGM-vegetation subgroups, with a target sample size of 2-3 per subgroup per stratum. The oversample size was 13 per stratum to accommodate any non- or negative-responses in the probability-selected sites. Note, that because of declined permissions for some legacy sites and several Category 1 random sites, it was necessary to do five in-kind substitutions to meet our target sample size. We ultimately requested permission to sample 89 sites, and were approved for 47 (although we sampled just 45). The 45 sites comprising the sample set spanned 16 counties in west–central Ohio (see Fig. 1 in the main document).

Section S2 : Confirmation of ORAM score and category

Many of the ORAM scores were outdated: e.g. most wetlands were scored over ten years ago, with some scored over twenty years ago. We updated all wetland ORAM scores and

categories using aerial imagery and notes from our field sampling. Specifically, we used current and historical imagery available through Google Earth to identify (1) any changes in wetland size, surrounding land use, and community interspersion; and (2) any large-scale disruptions to hydrology and habitat. During our site visits, we observed and recorded current site conditions: e.g. presence/absence of invasive plant species, disturbance of habitat or hydrology, presence of microtopographic features, quality of vegetation communities. Our reassessment also included querying Ohio's Natural Heritage Database (NHD). From the NHD, we received relevant documentation such as presence of critical habitat, presence of state or federal threatened and endangered species, and presence of significant breeding/nonbreeding bird concentration areas.

Typically, wetlands with an ORAM score of at least 60 are considered Category 3; wetlands with ORAM scores of at least 30, but less than 60 are considered Category 2; and wetlands with ORAM scores less than 30 are considered Category 1. Category assignment can be modified by additional criteria such as presence of threatened and endangered species or substantial presence of invasive plant species (Mack, 2001).

ORAM scores increased by an average of 3.7 points, with an absolute average increase of 5.2 points. The maximum increase was 28 points and the maximum decrease was 12 points. The revision of ORAM scores resulted in five formerly Category 1 wetlands being elevated to Category 2; six formerly Category 2 wetlands being elevated to Category 3; and one formerly Category 3 wetland being downgraded to Category 2.

With the re-assignment of ORAM categories, the distribution of wetlands across the three categories shifted (see Table 1 in the main document). The distribution of wetlands based on ORAM score, however, remained the same: e.g. ORAM score mean and range were similar between the original and current assessments.

Section S3 Soil sampling strategy

Soil samples were collected using a modified stratified random sampling strategy. Our sampling strategy for sample location balanced ease of sample collection, spatial representation for a limited number of samples per wetland, and inference strength. Ideally we would have implemented a stratified-random sampling design which as a probability-based method has high inference strength. This design also strategically locates random samples by strata and thus achieves greater accuracy in estimation of the population mean. The primary limitation to this design is that it requires more effort to implement (e.g. strata might be determined prior to sampling by an intensive vegetation survey; the survey data which is then used to identify strata based on vegetation communities; see e.g. Hossler et al., 2011).

Because we were sampling a large number of wetlands (45) over a short period of time (9 weeks), and because we wanted to follow a sampling protocol that would be time-efficient for "rapid" wetland assessments, we implemented a soil sampling strategy with aspects of both probability-based and non-probability-based sampling designs. The non-probability-based aspect was that we targeted five *a priori* zones (i.e. one per soil sample to be collected per wetland). This targeted sampling was based on elevational gradients present in most wetlands. These elevational gradients are known to create zones with characteristic hydrology, vegetation, and soil (Stewart and Kantrud, 1971; Millar, 1976; Cowardin et al., 1979; Reinecke et al., 2015; Daugherty et al., 2019). Stewart and Kantrud (1971), for example,

identified up to 5 zones within a wetland that had unique hydroperiods and vegetation communities. The probability-based aspect was the random selection of a transect pair within each wetland; the soil samples were then collected at fixed intervals along the transect pair.

Our strategy was essentially a form of systematic random sampling, which is a restricted random sampling procedure in which one typically samples *one* randomly selected cluster consisting of n subsamples. Conventionally in systematic random sampling, the subsamples are treated as independent random samples and both mean and variance are calculated using standard estimators based on simple random sampling. It is then accepted that the variance estimate will be biased and conservative (see Aune-Lundberg and Strand, 2014, and references therein for a discussion of caveats with systematic random sampling and alternative variance estimators).

Prior to each site visit, we first delineated wetland boundaries using recent and historical imagery available through Google Earth. When possible, these boundaries were based on National Wetland Inventory (NWI) polygon shapefiles. For wetlands that were more circular in shape, with edge-to-interior gradients (e.g. all depressional and some slope wetlands), two perpendicular lines radiating from the center of the wetland acted as the primary and secondary transects that intersected all five zones. The primary transects had three sample sites targeting the first, third, and fifth zone. The secondary transects had sample sites located in the second and fourth zones (Fig. S1a,b; see also Section S4). For wetlands that were more linear in shape, with upland-to-stream gradients (e.g. all riverine and some slope wetlands), two parallel lines that were perpendicular to the major wetland axis acted as the primary and secondary transects. Transect one had three sample sites targeting zones 1, 3, and 5. Transect two ran parallel at a predetermined distance from transect one and contained sample sites 2 and 4 (Fig. S1c,d).

Section S4 Sample selection for sites with edge-to-interior gradients

It was initially proposed that for determination of sampling stations within wetlands with edge-to-interior gradients (aka. circular type; e.g. all depressional and some slope wetlands), the center would be determined and the edge-to-center radius in a random direction (1 ° to 360 °) would be the first transect. Three sampling stations would be located at zone midpoints 1, 3 and 5 along the first transect (at $4.5\ell_1/5$, $2.5\ell_1/5$ and $0.5\ell_1/5$, where ℓ_1 is the length of transect 1); then two additional sampling stations would be located along a second transect, perpendicular to the first, at zone midpoints 2 and 4 (at $3.5\ell_2/5$ and $1.5\ell_2/5$, where ℓ_2 is the length of transect 2). This sampling strategy was selected to approximate a stratified sampling methodology based on observations that most wetlands have zones with characteristic hydrology, vegetation, and soil (Stewart and Kantrud, 1971; Millar, 1976; Cowardin et al., 1979; Reinecke et al., 2015; Daugherty et al., 2019). In circular-type wetlands, these zones arise from elevational gradients running edge-to-center. Our a priori division of five zones was based on Stewart and Kantrud (1971).

Two problems arising from our initial sampling strategy were 1) the wetland "center" might be outside of the wetland boundary depending on the estimator used and 2) selecting equidistant sampling station locations could miss expected zones because of unequal transect lengths. The next two subsections explore these issues; the third subsection details our solution to mitigate these issues.

Wetland center. It was desirable to have an automated method to locate wetland centers in our set of 45 wetland polygon shapefiles (and for future large-scale efforts). An obvious center-estimate is the center-of-mass centroid (aka. center-of-gravity or moment centroid) which is a very common center-estimator for polygons (Bourke, 1988; Bashein and Detmer, 1994; Deakin et al., 2002). The center-of-mass centroid for wetland polygon shapefiles can be easily calculated using the 'gCentroid' function in the R package *rgeos* (Bivand and Rundel, 2019). One problem with the center-of-mass centroid, however, is that for irregular wetlands, the centroid might be outside the wetland boundary (Farmer et al., 2011), or within the wetland boundary but very near the wetland edge (Fig. S2). Either scenario would make sample selection problematic. In the first case, all 360 possible transects radiating from the center-of-mass would be outside the wetland. In the second case, all transects would be inside the wetland but many would be very short leading to biased samples (i.e. a transect radiating toward the near boundary would result in samples along that transect being clustered at the boundary).

Zone representation. We initially proposed to select equally-spaced sampling stations along two perpendicular radial transects. This sampling design was intended to target up to five zones of characteristic hydrology, vegetation, and soil (e.g. Stewart and Kantrud, 1971; Millar, 1976; Cowardin et al., 1979; Reinecke et al., 2015; Daugherty et al., 2019). In nearly circular wetlands, this strategy should achieve one sampling station near the midpoint of each of the five anticipated zones (Fig. S3a). The zone boundaries are expected to occur at regular intervals along the edge-to-center radius. This expectation is based on observations of various ecological properties and processes that are spatially distributed by distance from an edge or boundary: e.g. seed dispersal (Cubiña and Aide, 2001; Ramsfield et al., 2020), soil and benthic fauna (Whaley and Minello, 2002; Riutta et al., 2016), soil chemistry (Schröder and Fleig, 2017; Barreto-Garcia et al., 2019), vegetation distribution (Barry et al., 2008; Ruwanza, 2019).

Misplaced wetland centers and irregular wetland boundaries can result in radial transects of varying lengths. The outcome being that equidistantly distributed sampling stations along the transects may not be located in the intended zone, with the outcome that certain targeted zones will be missed and others will be oversampled. This is particularly true for elongated and otherwise irregular wetlands (Fig. S3b,c). In Fig. S3b, for example, the wetland is elongated leading to shorter E–W transects and longer N–S transects; the problem is compounded because the center-of-mass centroid lies outside the innermost zone. For this wetland, the originally proposed sampling scheme would result in one sample each from zones 1, 2 and 5; 2 samples from zone 3; and no sample from zone 4.

A solution: pole-of-inaccessibility and polygon-reduction. To help mitigate these problems, we modified our sample selection of circular-type wetlands by

1. Estimating the wetland center using a pole-of-inaccessibility (POI) algorithm;

2. Estimating boundary locations for five hypothesized zones within each wetland using a polygon-reduction or negative-buffering algorithm;

3. Locating sampling stations at midpoints of the hypothesized zones along two perpendicular radial transects emanating from the POI (the first transect oriented in a random direction; i.e. 1 ° to 360 °).

First, we used the *pole-of-inaccessibility* or POI to estimate the wetland center. The POI is defined as the point most distant from all polygon edges (e.g. Garcia-Castellanos and Lombardo, 2007). Computation of the POI can be complex and time consuming, but a faster, approximating algorithm using quadtrees was recently developed by Agafonkin (2016). The algorithm is easily implemented in R using the 'poi' function in the R package *polylabelr* (Larsson, 2020). Unlike the center-of-mass centroid, the POI is always located within the wetland boundary (see e.g. Fig. S2).

Second, we estimated boundary locations for five hypothesized zones within each wetland using a polygon-reduction or negative-buffering algorithm. Polygon-reduction algorithms, such as that implemented in Farmer et al. (2011), sequentially shrink the polygon boundary by a fixed distance. This shrinking can be achieved with the 'gBuffer' function in the R package *rgeos* (Bivand and Rundel, 2019). Through this iterative procedure, polygon vertices trace the straight-line skeleton of the polygon (see e.g. Aichholzer et al., 1996; Farmer et al., 2011) and converge at a centerpoint (circular and other regular polygons) or centerline (elliptical and other irregular polygons). In our implementation, for a given wetland polygon shapefile, we first determined the edge-to-center distance (Δ_{ec}) using repeated calls to 'gBuffer' and very small negative increments. The five zone boundaries are then determined by calling 'gBuffer' at increments of $-\Delta_{ec}/5$, $-2\Delta_{ec}/5$, $-3\Delta_{ec}/5$, $-4\Delta_{ec}/5$ and $-\Delta_{ec}$ (e.g. this was the procedure used to generate the zone boundaries depicted in Figs. S2–S3). The rationale for this approach was the various observations of spatially-distributed ecological properties and processes relative to an edge or boundary (e.g. Cubiña and Aide, 2001; Barry et al., 2008; Riutta et al., 2016; Barreto-Garcia et al., 2019).

In the final step, similar to our initially proposed sampling strategy, we randomly selected an orientation (1 ° to 360 °) for the first radial transect. The transect emanated from the POI as determined in step 1. Along this first transect, sampling stations were located at midpoints for zones 1, 3 and 5, with the zones determined using the polygon-reduction method outlined for step 2. The second transect was oriented perpendicular to the first, with sampling stations located at midpoints for zones 2 and 4. Fig. S4 illustrates the end result for two of the wetlands depicted previously in Fig. S3. For both wetlands, the modified methodology correctly placed one sampling station in each of the five targeted zones, whereas the original methodology resulted in some zones being oversampled and some zones being missed.

To better assess the improvement in sample location provided by our modified sampling method we compared (1) center locations as estimated by either the center-of-mass centroid or the POI; and (2) zone representation as determined by the original method (using either the center-of-mass centroid or the POI as the center estimate) relative to the hypothesized zones (note, hypothesized zones were determined as described in step 2 and the modified sampling method by design always resulted in one sampling station per zone). We made these assessments for the subset of depression and slope wetlands in our ORAM dataset for which NWI polygon shapefiles were available (118 wetlands). Although the center-of-mass centroid algorithm only occasionally resulted in a center estimate outside of the wetland boundary (6 % of wetlands) or near the wetland boundary (13 % of wetlands), it frequently resulted in a center estimate outside of the wetland interior (as defined by zone 5; 47 % of wetlands). The POI algorithm improved the estimate of the wetland center by always lying within the wetland (i.e. 0 % external to the wetland) and only infrequently near the wetland edge (3 %

of wetlands) or outside of the wetland interior (12 % of wetlands). With respect to zone representation, the original sampling strategy using the center-of-mass centroid estimator resulted in at least one missed or oversampled zone in 72 % of the wetlands. The situation was somewhat improved by using instead the POI estimator, which resulted in only 41 % of the wetlands having a missed or oversampled zone by the original methodology. The best outcome was the modified sampling methodology, which by design resulted in exactly one sampling station per zone.

Section S5 Estimates of soil bulk density

Soil bulk density (ρ_b) is the soil dry mass per field-moist volume. There are various techniques to directly measure soil bulk density: e.g. core method, clod method, excavation method (Blake, 1965; Hao et al., 2007; Al-Shammary et al., 2018). In addition, some calculate ρ_b as the total mass per total volume (i.e. inclusion of all soil particles regardless of size). Others assess only the fine-particle (< 2 mm) fraction. Still others follow a hybrid approach and use the mass of the fine-particle fraction and the total volume (reviewed in Throop et al., 2012). We will refer to these three estimates of ρ_b as $\rho_{b,T}$, $\rho_{b,F}$ and $\rho_{b,H}$, respectively. The calculations are

$$\rho_{b,T} = \frac{M_{FF} + M_{CF}}{V_{FF} + V_{CF}} \tag{S1}$$

$$\rho_{b,F} = \frac{M_{FF}}{V_{FF}} \tag{S2}$$

$$\rho_{b,H} = \frac{M_{FF}}{V_{FF} + V_{CF}} \tag{S3}$$

where M_{FF} and M_{CF} are the dry masses of the fine-particle (< 2 mm) and coarse-particle ($\geq$ 2 mm) fractions of the soil; and V_{FF} and V_{CF} are the field-moist volumes of the fine-particle (< 2 mm) and coarse-particle ($\geq$ 2 mm) fractions of the soil. Typically, the fine-particle and coarse-particle fractions are separated by sieving after drying the soil. The mass of the coarse-particle fraction is then obtained and the volume is either measured by water displacement (e.g. Hao et al., 2007; Pearson et al., 2007; Throop et al., 2012; Mehler et al., 2014) or estimated using mean densities based on parent material (e.g. Federer et al., 1993; Maynard and Curran, 2007; Pearson et al., 2007; Mehler et al., 2014; Wade et al., 2020).

These three different calculations (i.e. Eqs. S1–S3) can result in vastly different estimates of ρ_b when the coarse-particle density differs substantially from the fine-particle density and the coarse-particle fraction is not negligible. For example, assume a fine-particle density of 0.7 g cm^{-3} and a coarse-particle density of 2.65 g cm^{-3} (e.g. gravel). If the coarse particles comprise 20% of the soil by volume, the resulting ρ_b calculations are 1.09, 0.70 and 0.56 g cm^{-3} for $\rho_{b,T}$, $\rho_{b,F}$ and $\rho_{b,H}$, respectively (Fig. S5a; see also Throop et al., 2012). If the coarse-particle density is 0.37 g cm^{-3} (e.g. coarse roots), the resulting ρ_b calculations are 0.63, 0.70 and 0.56 g cm^{-3} for $\rho_{b,T}$, $\rho_{b,F}$ and $\rho_{b,H}$, respectively (Fig. S5b). The relative difference between $\rho_{b,T}$ and $\rho_{b,F}$ (i.e. $\frac{(\rho_{b,T}-\rho_{b,F})}{\rho_{b,F}}$) is 0.56 in the first scenario and -0.09 in the second scenario (Fig. S6).

The method of ρ_b calculation is particularly important for properties that scale with a particular soil fraction. Estimates of carbon and nutrient stocks, for example, are determined primarily by the fine-particle fraction of the soil (e.g. Poeplau et al., 2017). Several studies have demonstrated substantial overestimation of carbon stocks when based on $\rho_{b,T}$ rather than $\rho_{b,F}$ or $\rho_{b,H}$ (Throop et al., 2012; Poeplau et al., 2017; Hobley et al., 2018). For example, assume the soil organic carbon (SOC) content is proportional to $\rho_{b,F}$ (as might be expected, see e.g. Poeplau et al., 2017). The coarse-particle fraction will determine $\rho_{b,T}$. If the coarse-particle fraction is constant, $\rho_{b,T}$ will have a similar relationship to SOC as $\rho_{b,F}$. If the coarse-particle fraction varies across samples, $\rho_{b,T}$ will be decoupled from SOC (Fig. S7).

Estimation of $\rho_{b,F}$ and $\rho_{b,H}$. Given the importance of bulk density calculation, we calculated all three metrics of bulk density (Eqs. S1–S3). Calculation of $\rho_{b,F}$ and $\rho_{b,H}$ required correction for the coarse-particle fraction (CF). The CF fraction includes coarse mineral matter (i.e. gravel) and coarse organic matter.

Coarse mineral matter was isolated from each core, weighed, and subjected to water displacement for volume estimation. The mass and volume were then subtracted appropriately. Coarse organic matter was corrected for by weighing for mass and then estimating volume using some previously determined value for coarse root bulk density (e.g. Federer et al., 1993; Wade et al., 2020). Federer et al. (1993) and Wade et al. (2020) use 0.7 g cm^{-3} and 0.2 g cm^{-3} for their estimates of organic matter (coarse root) bulk density, but provide no support for those values. We used an estimate of 0.37 g cm^{-3}, which is the coarse root bulk density based on root samples collected as part of the study by Hossler et al. (2011). In this study, root samples were collected from 0 to 30 cm depth from 15 emergent depressional wetlands in central Ohio. The estimate was based on 57 out of 236 samples for which the coarse root fraction (i.e. root diameter $\geq$ 2 mm) was at least 50% by volume. Root volume was estimated using WinRhizo software (Régent Instruments, Québec City, Quebec, Canada).

The Hossler et al. (2011) estimate of 0.37 g cm^{-3} is consistent with measurements by Bernier et al. (2005), who sampled roots to 25 cm depth in three Canadian forests. Root bulk densities averaged 0.34 g cm^{-3} to 0.49 g cm^{-3}. There was a strong size relationship for younger (e.g. seedling) roots: mean bulk densities were 0.20, 0.30 and 0.38 g cm^{-3} for root diameters of 1, 2 and 4 mm. Adams (1973) and De Vos et al. (2005) estimated the bulk density of soil organic matter to be 0.21–0.31 g cm^{-3} and 0.31 g cm^{-3}, respectively, by applying an organic matter and mineral mixing model to measurements of $\rho_{b,F}$ and organic matter content (mass-based) from forest soils. It should be noted that the estimates by Adams (1973) and De Vos et al. (2005) were based on soil and organic matter fractions $<$ 2 mm. Hence, their slightly lower estimates would be consistent with the size relationship observed by Bernier et al. (2005) (and Hossler et al., 2011, data not shown).

Section S6 Sensitivity Analysis

It should be noted, that the current 12-point Subetric 7a is conservative: e.g. under ORAM v6.0, maximum point contributions are 26 % for hydrology (Metric 3), 17 % for vegetation (Metric 6) and 16 % for soil (Metric 7). Obviously, higher weighting of the bulk-density based submetric (7a) would improve the representation of soil health. The point assignment for Submetric 7a could be increased to 13 to reach full equivalence with Metric 6 (i.e. both Metric 7 and Metric 6 contribute 17 % maximum) or increased to 20 to reach full equivalence

with Metric 3 (Fig. S8a). If Submetric 7a is increased to 13 points, the corresponding ORAM v6.0 scores increase slightly in correlation to soil health (PCA1; $r = 0.59$) and ρ_b ($r = -0.65$). An increase to 20 points for Submetric 7a decreases the correlation between ORAM v6.0 and ORAM v5.0 to $r = 0.97$, but increases the correlation of ORAM v6.0 with soil health and ρ_b to $r = 0.64$ and $r = -0.71$ (Fig. S8b; see also Table S1). The correlation of total ORAM scores to the individual metric scores could also be considered. For example, the original ORAM (v5.0) correlates strongly with Metrics 3 and 6 (both $r = 0.84$). The proposed 12-point Submetric 7a results in total ORAM (v6.0) correlations of $r = 0.75$ for Metric 7, $r = 0.82$ for Metric 3 and $r = 0.84$ for Metric 6. A 21-point Submetric 7a, results in equivalent correlation of Metrics 7 and 3 with ORAM v6.0 (both $r = 0.79$); whereas, a 28-point Submetric 7a, results in equivalent correlation of Metrics 7 and 6 with ORAM v6.0 (both $r = 0.82$; Fig. S8c).

Section S7 Reweighting Current ORAM v5.0 Metrics to Improve Correlation with Soil Health

In lieu of directly incorporating a soil-based metric into the current ORAM, we also explored reweighting of the current six ORAM v5.0 metrics to better reflect soil health. Constrained optimization was used to identify alternative point distributions for the current six ORAM metrics to better reflect soil health. Maximum point assignments under ORAM v5.0 are 6, 14, 30, 20, 10 and 20 for Metrics 1–6. We minimized the sum of square differences between the soil health score (PCA1, rescaled to range from 0 to 100) and a reweighted ORAM score, i.e. $\sum \mathrm{PCA1}_{100} - w^{\intercal}\mathrm{ORAM}$, where w is a vector of weights (one per metric) and were the parameters optimized over. These weights were initially set to 1 and were given a lower bound of 0. We used the "optim" function in R, with the L-BFGS-B method (a limited-memory quasi-Newtonian algorithm for bound-constrained optimization). For assessment of this method, we compared the correlation to overall soil health (PCA1), with the correlations of the current ORAM v5.0 and proposed ORAM v6.0 to overall soil health. We additionally applied repeated stratified k-fold cross validation, using 10 folds and 10 repeats. ORAM scores (v5.0, v6.0 and reweighted v5.0) were assessed based on how well they predicted PCA1 scores. We used the adjusted coefficient of determination as the assessment measure.

The optimized weights (rescaled so that maximum possible ORAM score remained 100) for current ORAM Metrics 1–6 (v5.0) were 6.5, 0, 0, 1.4, 0 and 1.6, respectively. Note, that in the reweighting, three metrics are excluded (i.e. weight = 0): Metrics 2, 3 (hydrology) and 5. The optimized weights yielded adjusted scores for the 45 wetlands ranging from 19 to 87; the range of their original ORAM v5.0 scores was 18 to 83. The correlation to soil health (PCA1) did improve from $r = 0.48$ using the original ORAM v5.0 point distributions to $r = 0.59$ using the reweighted point distributions. The improvement, however, was only achieved with complete exclusion of Metrics 2, 3 (hydrology) and 5. In contrast, incorporation of the conservative 12-point Metric 7a (bulk density) attained a similar correlation to soil health ($r = 0.58$) and retained all six original metrics.

Assessment by k-fold cross-validation supported a substantial improvement in correlation to overall soil health by reweighting the ORAM v5.0 scores ($R^2_{adj} = 0.29$) relative to the original ORAM v5.0 scores ($R^2_{adj} = 0.22$); but only modest improvement relative to the proposed ORAM v6.0 scores ($R^2_{adj} = 0.27$). Recall also, that the proposed ORAM v6.0 with

12 points assigned to Submetric 7a is somewhat conservative (e.g. see Fig. S8). Increasing the maximum points assigned to Submetric 7a increases the correlation with overall soil health: e.g. if assigned a maximum of 20 points (equal weighting with Metric 3), the correlation with overall soil health increases to $R^2_{adj} = 0.31$ (k-fold cross-validation).

Section S8 Recategorization based on ρ_b

One other alternative would be to recategorize wetlands based on ρ_b, through either 1) creating a narrative question or 2) using the "justification for recategorization" procedure in the current ORAM to assign Category 3 status (and potentially Category 2 status) to wetlands with mean soil ρ_b above a certain threshold. For the latter, the final question of the "Wetland Categorization Worksheet" of ORAM v5.0 asks the rater "Does the wetland otherwise exhibit *moderate OR superior* hydrologic OR habitat, OR recreational functions AND the wetland was *not* categorized as a Category 2...or a Category 3 wetland...?" A response of "YES" indicates that "Wetland was undercategorized by this method. A written justification for recategorization should be provided...." (see final page of Mack, 2001). If we consider "habitat" to be inclusive of soil, then this would be an existing mechanism to elevate the status of wetlands with high quality soils.

Adding a narrative question would be a way to formalize this recategorization. ORAM v5.0 has eleven narrative questions(two are multipart)—eight of which can result in assignment to Category 3. For example, wetlands containing threatened or endangered species, or considered bogs or fens or "old growth forest" would be assigned Category 3 if the ORAM score was at least 35. If the ORAM score is less than 35, an assignment of Category 3 is still possible; however, the rater is referred to Ohio Administrative Code (OAC) Rule 3745-1-54(C) before making the final determination. (Note that, typically an ORAM score less than 30 results in an assignment of Category 1; ORAM scores of 30 to 34.9 are in the Category 1/2 gray zone, which generally defaults to placement in the higher category.)

Similarly, a narrative question for soil quality could be used to assign Category 3 or Category 2 to a wetland based on its mean ρ_b. We recommend that wetlands with mean bulk densities < 0.40 g cm^{-3} be delegated Category 3, and wetlands with mean bulk densities ≤ 0.73 g cm^{-3}, be delegated Category 2 or higher (Fig. S9a). Additionally, points could be awarded based on ρ_b using the existing Metric 5 (with modification). Metric 5 ("Special Wetland Communities") was added in v5.0 of the ORAM to help quantify the Narrative Rating section by either adding points or deducting points based on the narrative questions (Mack, 2001). In accordance with the existing options in Metric 5, we would suggest awarding 5 points if $\rho_b \leq 0.73$ g cm^{-3} but ≥ 0.40 g cm^{-3}; and 10 points if $\rho_b < 0.40$ g cm^{-3} (Fig. S9b). Either of these two options would enable increasing the valuation of the soil without detracting from other components. The proposed revision to Metric 5, for example, would result in the upgrading of two Category 1 wetlands to Category 2 and two Category 2 wetlands to Category 3 based on soil condition.

Section S9 Estimation of Soil Texture

Soil texture was obtained from the USDA Soil Survey Geographic (SSURGO) Database (Soil Survey Staff, 2024). Soil survey map units associated with each wetland sample location were

identified by latitude and longitude using the packages *rgdal* and *rgeos* in R. Each soil map unit consisted of one or more soil components, and each each soil component consisted of multiple soil horizons. Map units were identified by 'mukey' and ultimately linked to a 'chkey' (i.e. a unique identifier for a soil horizon within a map unit soil component) through a series of tabular files (e.g. *mapunit*, *comp*, *chorizon*). The 'chkey' enabled extraction of percent sand, silt and clay; soil texture class; and other soil properties for each map unit soil horizon associated with a sample location.

For each wetland sample, associated SSURGO soil properties were first weight-averaged as needed across soil horizons within each soil component. Horizons were combined to a depth of 15 cm (the depth of the collected soil cores). For example, if the top horizon had a thickness of 10 cm, the weight-averaged soil property would be $(10 \times prop_1 + 5 \times prop_2)/15$, where $prop_1$ is the soil property of the first (top) horizon and $prop_2$ is the soil property of the second horizon. Weight-averaged soil properties were then combined across map unit components based on their relative contributions. Only major components were considered. For example, if one major component comprised 50% of the map unit and a second major component comprised 45% of the map unit, the weight-averaged soil property would be $(50 \times prop_1 + 45 \times prop_2)/95$, where $prop_1$ is the weight-average soil property across horizons for the first component and $prop_2$ is the weight-averaged soil property across horizons for the second component. Note, 33 wetland samples were identified in the SSURGO Database as muck, water, or Udorthents. These map units lacked most soil properties. For these samples, soil properties were estimated based on nearby map units.

Following the above procedure, we estimated the following SSURGO-based soil properties for each wetland sample: sand (%), silt (%), clay (%), organic matter (g hg^{-1}) and bulk density at one-third bar tension (g cm^{-3}). The proportions of sand, silt and clay were used to 1) identify the USDA soil texture class for the weight-averaged estimates of sand, silt, and clay; and 2) calculate the geometric mean particle diameter (d_g; range is 0.001 mm for all clay to 1.025 mm for all sand). Using the formula in (Römkens et al., 1997), d_g was calculated as $\exp(0.01 \sum f_i \ln m_i)$, where f_i and m_i are the proportions and mean diameters of sand, silt and clay (mean diameters were estimated as 1.025 mm, 0.026 mm, and 0.001 mm, respectively).

Supplementary Tables

Table S1: The points assigned to the proposed soil bulk density Metric (7a) altered the ORAM v6.0 scores and the correlation of ORAM v6.0 with the original ORAM scores (v5.0), soil health (PCA1) and ρ_b.

Metric 7a	ORAM v6.0 Correlation			
points	v5.0	PCA1	ρ_b	Comment
0	1.00	0.48	-0.53	original ORAM v5.0 score
12	0.99	0.58	-0.64	first proposed point assignment
13	0.99	0.59	-0.65	equal weighting of Metric 7 and Metric 6
20	0.97	0.64	-0.71	equal weighting of Metric 7 and Metric 3
21	0.97	0.65	-0.72	equal correlation of ORAM v6.0 with Metrics 7 and 3
28	0.94	0.70	-0.77	equal correlation of ORAM v6.0 with Metrics 7 and 6

Table S2: Overall soil health based on ρ_b, $SoilC$ and $MWDc$ was compared to ORAM score with inclusion of either hydrogeomorphic class (HGM) or dominant vegetation (VEG) as a covariate. The relationships were assessed by PERMANOVA and the results are reported for ORAM score and the interaction of ORAM score with the covariate. When the three potential outliers were removed, the interaction terms became statistically insignificant.

	ORAM			ORAM:covariate		
model	*df*	*F*	*p*	*df*	*F*	*p*
**full dataset**						
$ORAMv5 + ORAMv5 : VEG$	1,41	12.5	0.001	2,41	6.90	0.001
$ORAMv6 + ORAMv6 : VEG$	1,41	20.3	0.001	2,41	5.97	0.002
$ORAMv5 + ORAMv5 : HGM$	1,41	11.4	0.001	2,41	4.54	0.010
$ORAMv6 + ORAMv6 : HGM$	1,41	18.7	0.001	2,41	3.91	0.010
**three outliers removed**						
$ORAMv5 + ORAMv5 : VEG$	1,38	22.7	0.001	2,38	2.37	0.067
$ORAMv6 + ORAMv6 : VEG$	1,38	30.7	0.001	2,38	1.95	0.129
$ORAMv5 + ORAMv5 : HGM$	1,38	20.9	0.001	2,38	0.68	0.588
$ORAMv6 + ORAMv6 : HGM$	1,38	28.9	0.001	2,38	0.72	0.537

Table S3: The soil health index (PCA1) was compared to ORAM score with inclusion of either hydrogeomorphic class (HGM) or dominant vegetation (VEG) as a covariate. The relationships were assessed by simple linear regression and the results are reported for ORAM score and the interaction of ORAM score with the covariate. When the three potential outliers were removed, the interaction terms became statistically insignificant.

	ORAM			ORAM:covariate		
model	*df*	*F*	*p*	*df*	*F*	*p*
**full dataset**						
$ORAMv5 + ORAMv5 : VEG$	1,41	18.4	<0.001	2,41	10.1	<0.001
$ORAMv6 + ORAMv6 : VEG$	1,41	31.2	<0.001	2,41	8.99	<0.001
$ORAMv5 + ORAMv5 : HGM$	1,41	16.0	<0.001	2,41	6.23	0.004
$ORAMv6 + ORAMv6 : HGM$	1,41	27.4	<0.001	2,41	5.42	0.008
**three outliers removed**						
$ORAMv5 + ORAMv5 : VEG$	1,38	36.3	<0.001	2,38	3.13	0.055
$ORAMv6 + ORAMv6 : VEG$	1,38	52.2	<0.001	2,38	2.54	0.093
$ORAMv5 + ORAMv5 : HGM$	1,38	32.2	<0.001	2,38	0.61	0.550
$ORAMv6 + ORAMv6 : HGM$	1,38	47.6	<0.001	2,38	0.60	0.552

Supplementary Figures

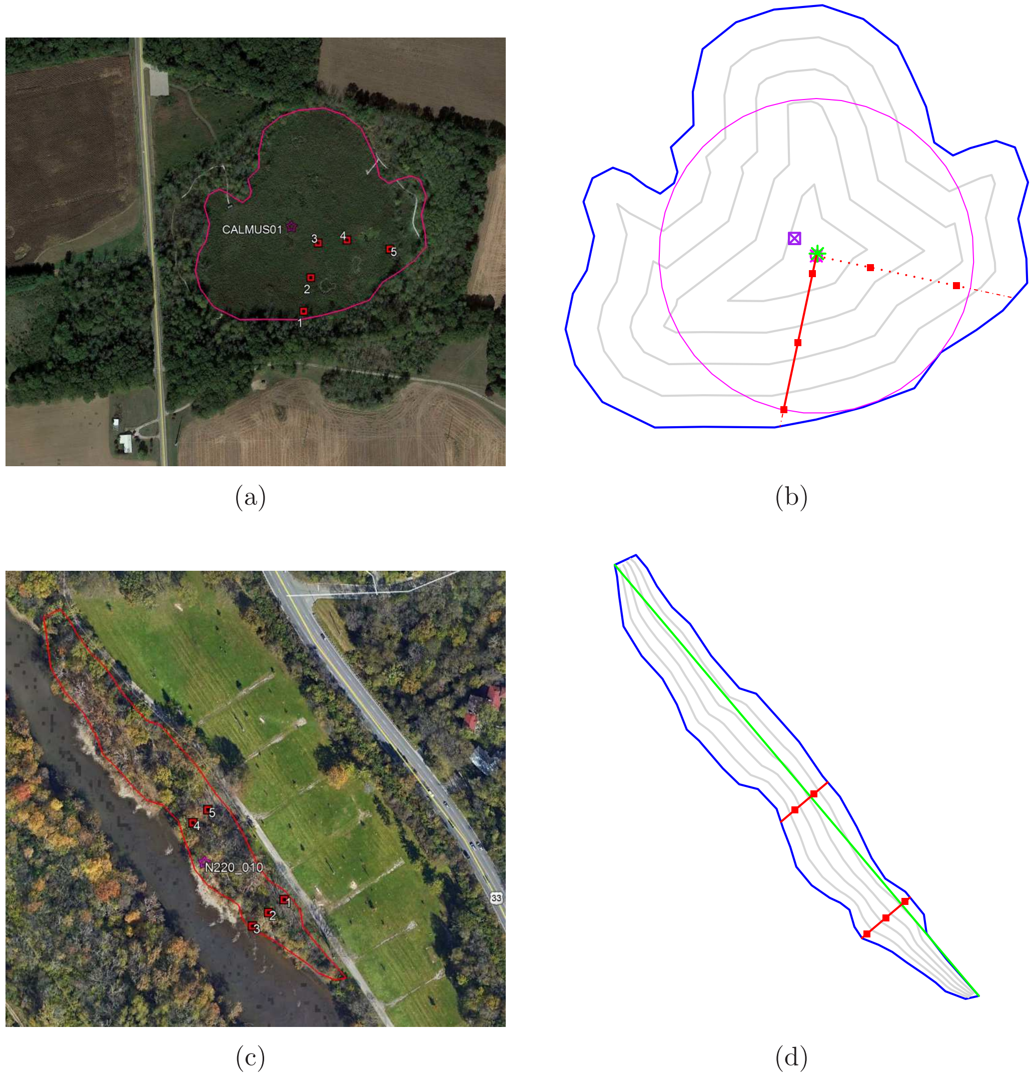

Figure S1: Example sampling schemes for wetlands with an edge-to-interior gradient (a,b) and wetlands with an upland-to-stream gradient (c,d). In each image pair, the image on the left displays the aerial imagery with sample locations identified; the image on the right depicts the zone boundaries, transects and sample locations.The general strategy was to sample from each of five *a priori* zones, that based on underlying gradients, would have characteristic hydrology, vegetation, and soil.

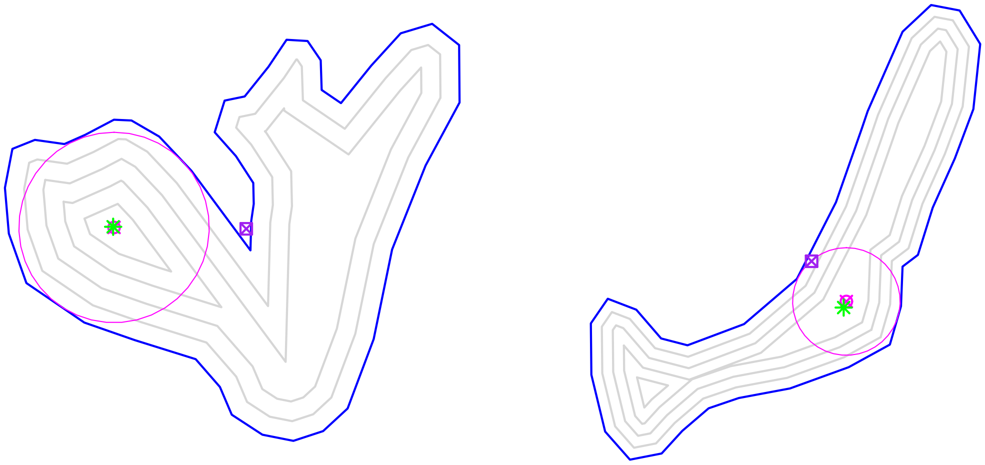

(a) (b)

Figure S2: Select wetlands demonstrating that the center-of-mass centroid (purple square with cross) can be outside of the wetland boundary (a) or within the wetland but near the wetland boundary (d). The pole-of-inaccessibility or POI (magenta circle with cross) is much more robust to this problem and always lies within the wetland. For further comparison, the center-of-mass centroid estimated from the wetland interior polygon (innermost polygon) is also depicted (green asterisk). [The wetland boundary is indicated by the blue line and rings demarcating the five zone boundaries are indicated by the gray lines (note, that some zones are not depicted at the rendered scale).]

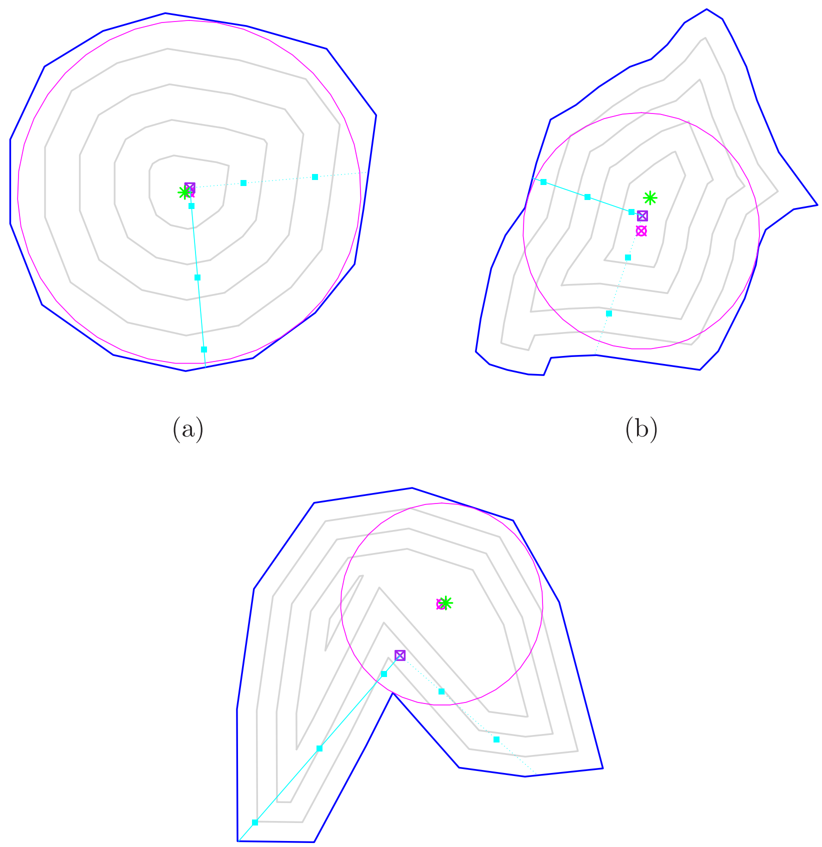

(a) (b) (c)

Figure S3: In (a), for approximately circular wetlands the initial sampling methodology is sufficient to achieve sampling stations (cyan squares) located near the midpoints of zones 1–5. In (b) and (c), for more irregularly-shaped wetlands the initial sampling methodology fails to locate sample locations properly (i.e. not in the targeted zones). In (b), the irregularity of the wetland boundary produces longer or shorter radial transects depending on orientation. In (c), the problem is compounded because the center-of-mass centroid (purple square with cross) lies outside the innermost zone. In the (b) and (c) examples, the equidistantly spaced sampling stations miss some targeted zones and oversample others. In (a), for example, the proposed sampling scheme would result in one sample each from zones 1–3, two samples from zone 5, and no sample from zone 4. [The wetland boundary is indicated by the blue line and rings demarcating the five zone boundaries are indicated by the gray lines. Three center points are also indicated: center-of-mass centroid for the entire wetland, purple square with cross; pole-of-inaccessibility, magenta circle with cross; and center-of-mass centroid for the wetland interior (innermost polygon), green asterisk.]

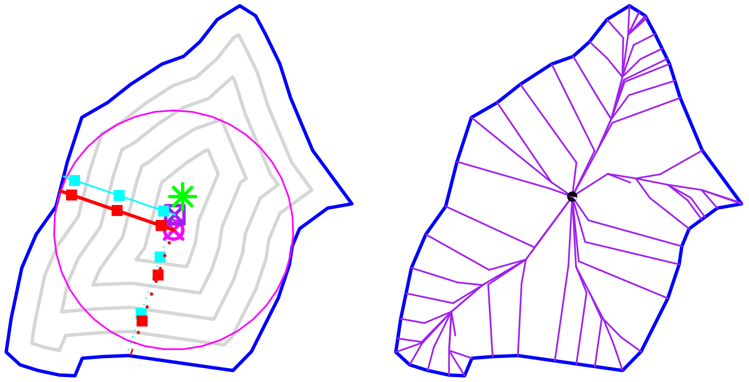

(a)

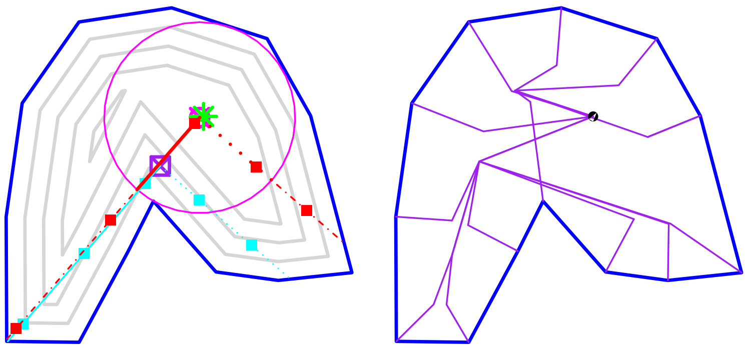

(b)

Figure S4: Sampling station location is improved by our modification to the original sampling scheme. We illustrate this improvement with two wetlands depicted in Fig. S3b,c. For both wetlands, radial transects and sampling station locations determined by the modified methodology are depicted with red lines and squares. For comparison, radial transects and sampling station locations determined by the original methodology are depicted with cyan lines and squares. In (a), corresponding to Fig. S3bb, the modified strategy correctly places one sampling station in each of the five targeted zones; whereas in the original methodology, zone 5 was oversampled and zone 4 was missed. In (b), corresponding to Fig. S3c, the modified strategy again correctly places one sampling station in each of the five targeted zones; whereas in the original methodology, zones 1 and 2 were oversampled and zones 4 and 5 were missed. [The wetland boundary is indicated by the blue line and rings demarcating the 5 zone boundaries are indicated by the gray lines. Three center points are also indicated: center-of-mass centroid for the entire wetland, purple square with cross; pole-of-inaccessibility, magenta circle with cross; and center-of-mass centroid for the wetland interior (innermost polygon), green asterisk. Also depicted are the approximate straight-line skeletons for each wetland (right subfigures)—the paths traced by the vertices during polygon reduction.]

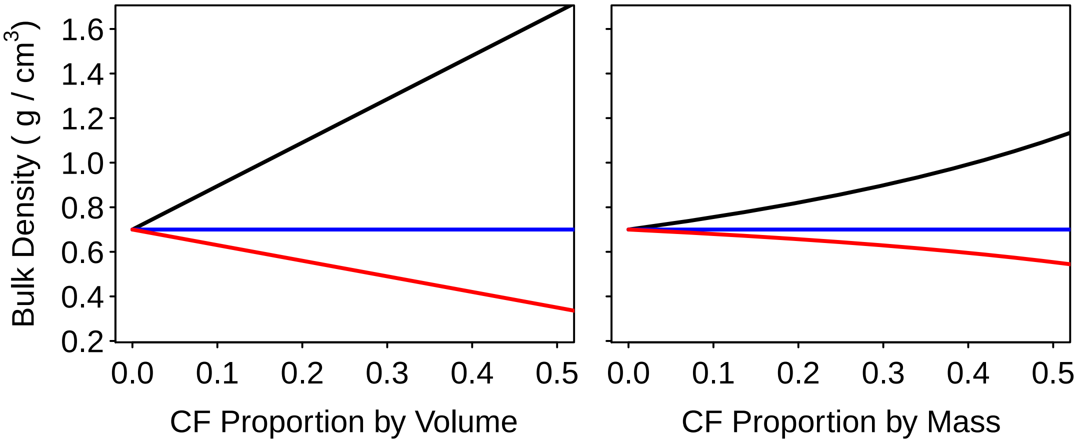

(a)

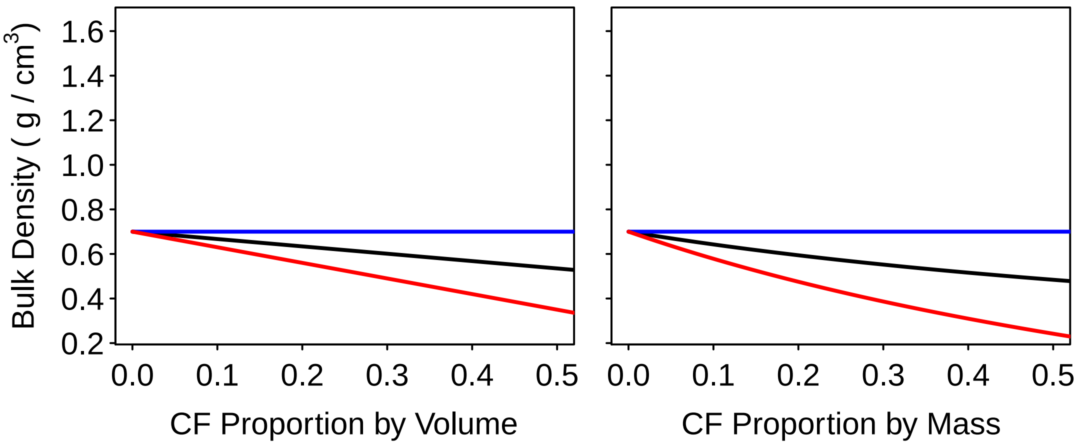

(b)

Figure S5: Three different calculations of ρ_b with respect to the coarse-particle (CF) proportion by volume and by mass. In the first method of calculation all soil is used to estimate ρ_b (black line; $\rho_{b,T}$). In the second method, only the fine-particle fraction is used to estimate ρ_b (blue line; $\rho_{b,F}$). In the third method, the calculation uses the fine-particle mass and the total soil volume (red line; $\rho_{b,H}$). In (a), the CF density is assumed to be 2.65 g cm^{-3}, i.e. gravel. In (b), the CF density is assumed to be 0.37 g cm^{-3}, i.e. coarse roots. In all cases, the fine-particle density is assumed to be 0.7 g cm^{-3}.

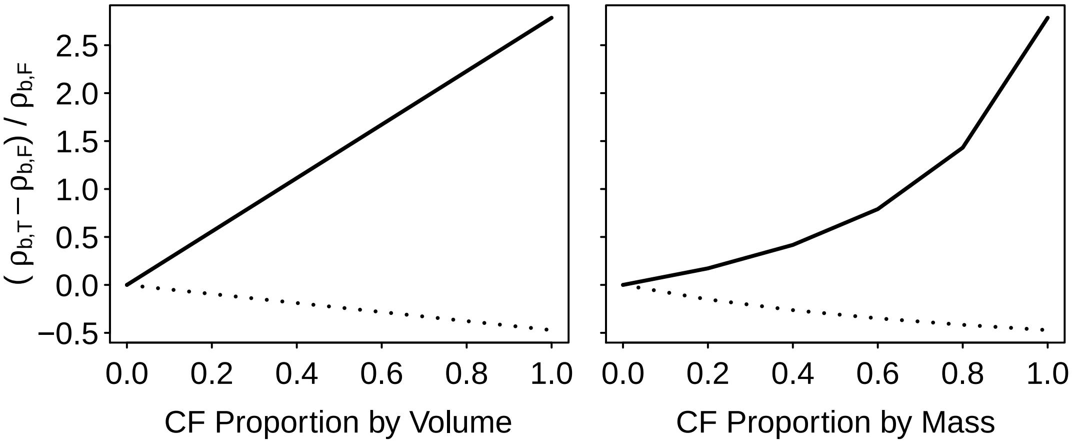

Figure S6: The relative difference between $\rho_{b,T}$ and $\rho_{b,F}$ given the proportion of coarse-particles (CF) by volume or mass. The fine-particle density was assumed to be 0.7 g cm^{-3} and the coarse-particle density was modeled as either 2.65 g cm^{-3} (solid line; e.g. gravel) or 0.37 g cm^{-3} (dotted line; e.g. coarse roots). (The 0.37 g cm^{-3} coarse root density was based on root samples collected as part of the study by Hossler et al. (2011). Root samples were collected from 0 to 30 cm depth. The estimate was based on 57 out of 236 samples for which the coarse root fraction (i.e. ≥ 2 mm) was at least 50% by volume.)

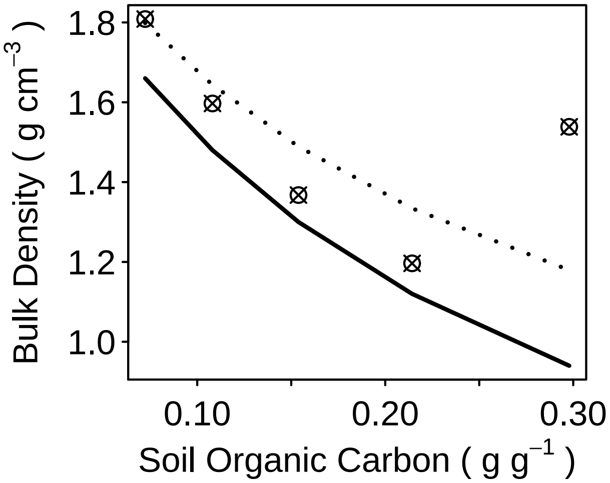

Figure S7: Variable amounts of coarse-particles will impact the relationship between $\rho_{b,T}$ and soil organic carbon (SOC). In this example, a simple mixing model was used to relate the fine-particle bulk density ($\rho_{b,F}$) to SOC (g g^{-1}): $\rho_{b,F} = \frac{f_o \rho_o}{SOC}$, where f_o is the volumetric proportion of organic carbon in the fine-particle fraction and ρ_o is the bulk density of the organic carbon fraction. The solid black line indicates the modeled $\rho_{b,F}$ and SOC; the correlation for this simulated sample was $r = -0.98$. If the coarse-particle fraction is constant across all samples, $\rho_{b,T}$ will also be strongly correlated to SOC ($r = -0.98$, with $f_o = 0.14$; dotted black line). When the coarse-particle fraction is variable, $\rho_{b,T}$ and SOC become uncoupled ($r = -0.51$; circled crosses).

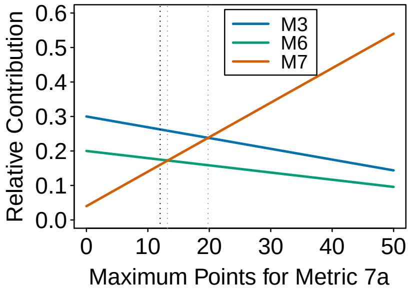

(a)

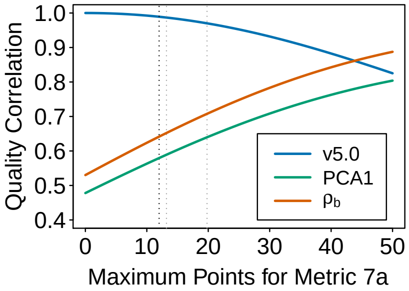

(b)

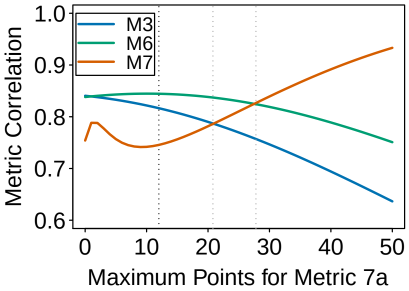

(c)

Figure S8: In (a), increasing the weight of Submetric 7a increases the relative contribution of Metric 7 (M7) and decreases the relative contributions of Metric 3 (M3) and Metric 6 (M6) to the maximum ORAM score. In (b), increasing the weight of Submetric 7a decreases the correlation between ORAM v6.0 and ORAM v5.0 scores, but increases the correlation of ORAM v6.0 scores with soil health (PCA1) and ρ_b. In (c), increasing the weight of Submetric 7a increases the correlation of ORAM v6.0 scores with Metric 7, but decreases the correlation of ORAM v6.0 with Metrics 3 and 6. In all subfigures, the vertical black dotted line indicates the proposed weight of Submetric 7a of 12 points. In (a) and (b), the two vertical gray dotted lines indicate potential weights of 13 points (to reach full equivalence with Metric 6) and 20 points (to reach full equivalence with Metric 3). In (c), the two vertical gray dotted lines indicate potential weights of 21 points (to reach ORAM v6.0 correlation strength equal with Metric 3) and 28 points (to to reach ORAM v6.0 correlation strength equal with Metric 6). (Note, the unusual pattern for Metric 7 in (c) is because ORAM v5.0 correlates more strongly with with Submetric 4a ($r = 0.75$)—which becomes Submetric 7b in ORAM v6.0—than with the proposed Submetric 7a ($r = 0.54$); and because the calculated scores for ORAM v6.0 and Metric 7 are both changing based on the Submetric 7a point assignment.)

**12a**	**High Quality Soil.** Does the wetland contain high quality soil, that is characterized by a mean bulk density of < 0.40 g cm^{-3} (based on soil cores collected to 15 cm depth)?	YES<br>Wetland is a Category 3 wetland<br>Complete Quantitative Rating	NO<br>Go to Question 12b
**12b**	Does the wetland contain high quality soil, that is characterized by a mean bulk density of ≤ 0.73 g cm^{-3} (based on soil cores collected to 15 cm depth)?	YES<br>Wetland is a Category 2 wetland or higher.<br>Complete Quantitative Rating	NO<br>Complete Quantitative Rating

(a)

Metric 5. Special Wetlands.

max 10 pts. subtotal

Check all that apply and score as indicated.

- Bog (10)
- Fen (10)
- Old growth forest (10)
- Mature forested wetland (5)
- Lake Erie coastal/tributary wetland-unrestricted hydrology (10)
- Lake Erie coastal/tributary wetland-restricted hydrology (5)
- Lake Plain Sand Prairies (Oak Openings) (10)
- Relict Wet Prairies (10)
- Known occurrence state/federal threatened or endangered species (10)
- Significant migratory songbird/water fowl habitat or usage (10)
- **Mean soil bulk density < 0.40 g cm^{-3} (10)**
- **Mean soil bulk density ≥ 0.40 and ≤ 0.73 g cm^{-3} (5)**
- Category 1 Wetland. See Question 1 Qualitative Rating (-10)

(b)

Figure S9: Alternatively, a narrative question for wetland soil condition could be added to ORAM v5.0 as in (a). This question could also be quantified in Metric 5 of the ORAM as shown in (b). This would leave the current ORAM v5.0 scoring system intact, but provide a formal mechanism to protect wetlands with high quality soils.

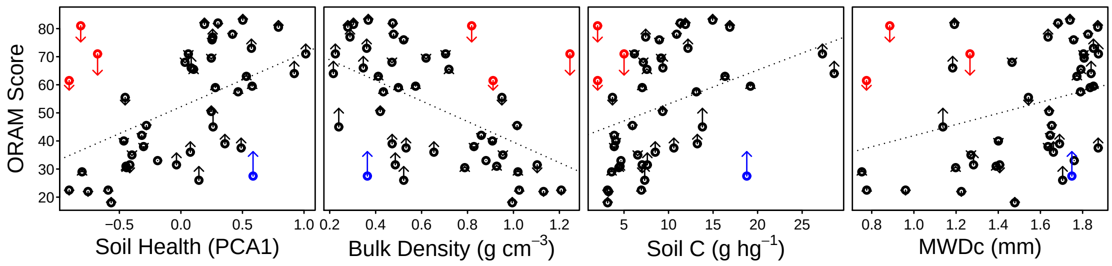

Figure S10: Relationships between ORAM score with (a) overall soil health and the three soil quality indicators (b) ρ_b, (c) $SoilC$, and (d) $MWDc$. Relationships are plotted using the original ORAM scores (v5.0; points) and revised ORAM scores (v6.0; arrow tip). Three wetlands had high ORAM scores but relatively low soil health (red); one wetland had a low ORAM score but relatively high soil health (blue). (These four wetlands are among the six potential outliers noted in the main document; e.g. Fig. 3.)

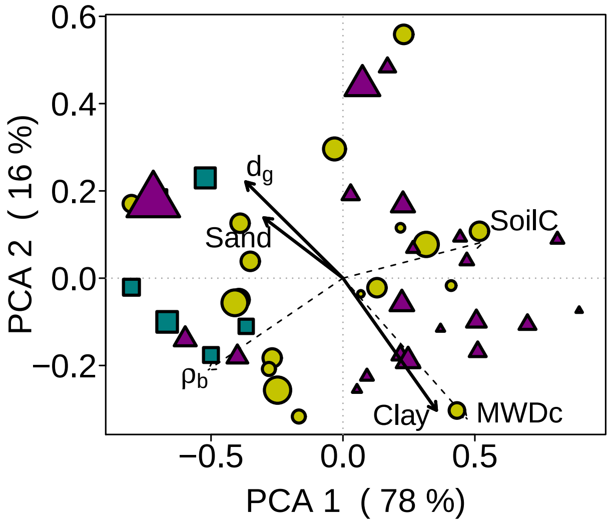

Figure S11: Principal component analysis (PCA) was applied to the three measurements of soil health. Overall soil health correlated with the geometric mean particle diameter (d_g, log transformed; $F_{1,43} = 9.40$, $R^2 = 0.18$, $p = 0.001$); percent sand ($F_{1,43} = 5.57$, $R^2 = 0.11$, $p = 0.016$); and percent clay ($F_{1,43} = 9.26$, $R^2 = 0.18$, $p = 0.002$). There was no significant correlation with percent silt. Points are shaped and colored by ORAM v5.0 category (green squares, Category 1; yellow circles, Category 2; purple triangles, Category 3) and sized by d_g. The figure also shows the projections of d_g (log transformed), percent sand, and percent clay onto the PCA ordination (note, the projections were rescaled to better fit in the current ordination). Statistical relationships were assessed by PERMANOVA.

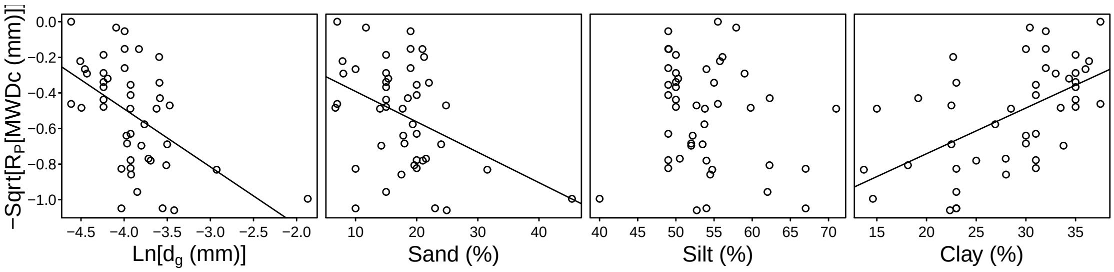

(a)

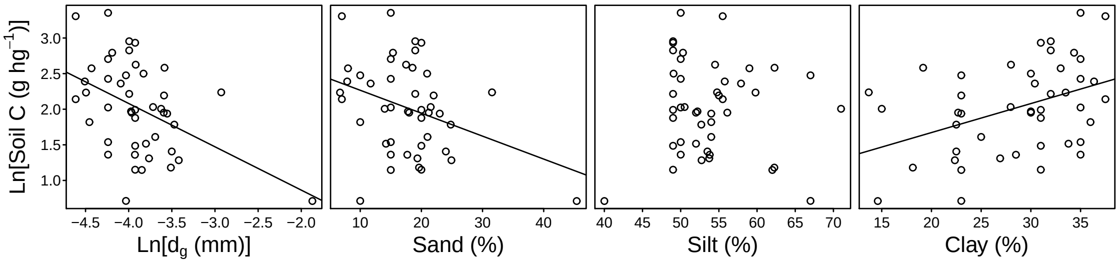

(b)

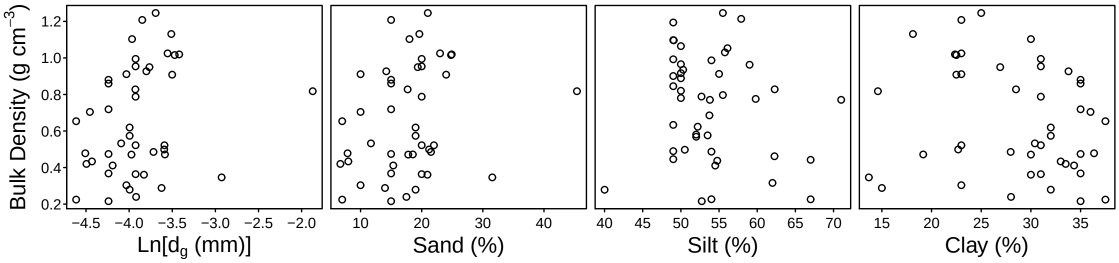

(c)

Figure S12: Relationships between overall soil health and soil texture (e.g. Fig. S11) were driven by relationships between the soil quality indicators (a) $MWDc$ and (b) $SoilC$ with geometric mean particle diameter (d_g; $R^2 = 0.27$ and $R^2 = 0.20$), percent sand ($R^2 = 0.16$ and $R^2 = 0.12$), and percent clay ($R^2 = 0.32$ and $R^2 = 0.17$). These relationships resulted in statistical significance at $p \leq 0.05$, with $n = 43$ degrees of freedom. The other relationships were not statistically significant—including all relationships of (c) soil bulk density with soil texture. (To meet assumptions of linearity, $SoilC$ and d_g were log transformed; $MWDc$ was reflect square root transformed then multiplied by -1 to preserve order.)

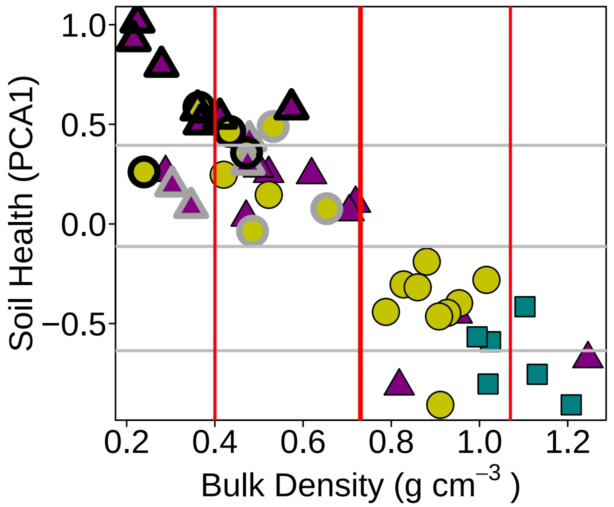

Figure S13: Wetlands with organic-dominated soils are indicated on this plot of soil health score (PCA1; y-axis) and ρ_b (x-axis). The criterion for "organic soil" is $SoilC \geq 12$ (Soil Survey Staff, 2022). Eleven wetlands met the criterion on a per-wetland basis (i.e. based on weighted averages; outlined in black). An additional seven wetlands met the criterion on a per-soil basis (i.e. at least one soil sample from the wetland was organic; outlined in gray). [The vertical red lines indicate the recommended bin break points: 0.40, 0.73 and 1.07 g cm^{-3}. The horizontal gray lines indicate the approximate corresponding soil health break points: 0.39, -0.11, -0.63. Wetlands are shaped and colored by revised ORAM (v6) category: green squares, Category 1; yellow circles, Category 2; purple triangles, Category 3. This figure is referenced in "Organic soil material" in the main document.]

Supplementary References

Aichholzer, O., Aurenhammer, F., Alberts, D., Gärtner, B.: A novel type of skeleton for polygons. In: Maurer, H., Calude, C., Salomaa, A. (eds.) J.UCS The Journal of Universal Computer Science. Springer, Heidelberg, Germany (1996). https://doi.org/10.1007/978-3-642-80350-5_65

Adams, W.A.: The effect of organic matter on the bulk and true densities of some uncultivated podzolic soils. European Journal of Soil Science **24**, 10–17 (1973) https://doi.org/10.1111/j.1365-2389.1973.tb00737.x

Agafonkin, V.: A new algorithm for finding a visual center of a polygon. [Available at https://blog.mapbox.com/a-new-algorithm-for-finding-a-visual-center-of-a-polygon-7c77e6492fbc] (2016)

Aune-Lundberg, L., Strand, G.-H.: Comparison of variance estimation methods for use with two-dimensional systematic sampling of land use/land cover data. Environmental Modeling and Software **61**, 87–97 (2014) https://doi.org/10.1016/j.envsoft.2014.07.001

Al-Shammary, A.A.G., Kouzani, A.Z., Kaynak, A., Khoo, S.Y., Norton, M., Gates, W.: Soil bulk density estimation methods: a review. Pedosphere **28**, 581–596 (2018) https://doi.org/10.1016/S1002-0160(18)60034-7

Barry, M.J., Andreas, B.K., De Szalay, F.A.: Long-term plant community changes in managed fens in Ohio, USA. Aquatic Conservation: Marine and Freshwater Ecosystems **18**, 392–407 (2008) https://doi.org/10.1002/aqc.832

Bashein, G., Detmer, P.R.: I.1 Centroid of a polygon. In: Heckbert, P.S. (ed.) Graphics GEMS IV. The Graphics Gems Series: A collection of Practical Techniques for the Computer Graphics Programmer, pp. 3–6. AP Professional, Cambridge, MA, USA (1994)

Barreto-Garcia, P.A.B., Scoriza, R.N., Paula, A.: Edge effect on chemical attributes of soil in a seasonal forest. Revista Árvore **43**, 430109 (2019) https://doi.org/10.1590/1806-90882019000100009

Blake, G.R.: Bulk density. In: Black, C.A. (ed.) Methods of Soil Analysis, Part 1, pp. 374–390. American Society of Agronomy, Inc., Madison, WI (1965)

Bourke, P.: Calculating the area and centroid of a polygon. [Available at http://paulbourke.net/geometry/polygonmesh/] (1988)

Bivand, R., Rundel, C.: Rgeos: Interface to Geometry Engine – Open Source (‘GEOS’). (2019). R package version 0.5–2, [Available at https://CRAN.R-project.org/package=rgeos]

Bernier, P.Y., Robitaille, G., Rioux, D.: Estimating the mass density of fine roots of trees

for minirhizotron-based estimates of productivity. Canadian Journal of Forest Research **35**, 1708–1713 (2005) https://doi.org/10.1139/x05-099

Cubiña, A., Aide, T.M.: The effect of distance from forest edge on seed rain and soil seed bank in a tropical pasture. Biotropica **33**, 260–267 (2001) https://doi.org/10.1111/j.1744-7429.2001.tb00177.x

Cowardin, L.M., Carter, V., Golet, F.C., LaRoe, E.T.: Classification of Wetlands and Deepwater Habitats of the United States vol. FWS/OBS-79/31. U.S. Fish and Wildlife Service, Washington, D.C. (1979)

Deakin, R.E., Bird, S.C., Grenfell, R.I.: The centroid? where would you like it to be be? Cartography **31**, 153–167 (2002) https://doi.org/10.1080/00690805.2002.9714213

Daugherty, E.E., McKee, G.A., Bergstrom, R., Burton, S., Pallud, C., Hubbard, R.M., Kelly, E.F., Rhoades, C.C., Borch, T.: Hydrogeomorphic controls on soil carbon composition in two classes of subalpine wetlands. Biogeochemistry **145**, 161–175 (2019)

De Vos, B., Van Meirvenne, M., Quataert, P., Deckers, J., Muys, B.: Predictive quality of pedotransfer functions for estimating bulk density of forest soils. Soil Science Society of America Journal **69**, 500–510 (2005) https://doi.org/10.2136/sssaj2005.0500

Farmer, E., Jones, S.D., Deakin, R.E.: Derivation of a resilient polygon centroid for natural resource management applications. International Journal of Geographical Information Science **25**, 1545–1558 (2011) https://doi.org/10.1080/13658816.2011.554837

Federer, C.A., Turcotte, D.E., Smith, C.T.: The organic fraction – bulk density relationship and the expression of nutrient content in forest soils. Canadian Journal of Forest Research **23**, 1026–1032 (1993)

Garcia-Castellanos, D., Lombardo, U.: Poles of inaccessibility: A calculation algorithm for the remotest places on earth. Scottish Geographical Journal **123**, 227–233 (2007) https://doi.org/10.1080/14702540801897809

Gara, B., Schumacher, B.: Intensification of the National Wetland Condition Assessment for Ohio: Final report. Ohio EPA Technical Report WET/2015-1, Ohio Environmental Protection Agency, Wetland Ecology Group, Division of Surface Water, Columus, Ohio, USA (2015)

Hao, X., Ball, B.C., Culley, J.L.B., Carter, M.R., Parkin, G.W.: Soil density and porosity. In: Carter, M.R., Gregorich, E.G. (eds.) Soil Sampling and Methods of Analysis, pp. 743–759. CRC Press, Taylor and Francis Group, Boca Raton, FL, USA (2007). Chap. 57

Hossler, K., Bouchard, V., Fennessy, M.S., Frey, S.D., Anemaet, E., Herbert, E.: No-net-loss not met for nutrient function in freshwater marshes: recommendations for wetland mitigation policies. Ecosphere **2**, 82 (2011) https://doi.org/10.1890/ES11-00009.1

Hobley, E.U., Murphy, B., Simmons, A.: Comment on "Soil organic stocks are systematically overestimated by misuse of the parameters bulk density and rock fragment content" by Poeplau et al. (2017). SOIL **4**, 169–171 (2018) https://doi.org/10.5194/soil-4-169-2018

Larsson, J.: Polylabelr: Find the Pole of Inaccessibility (visual Center) of a Polygon. (2020). R package version 0.2.0, [Available at https://CRAN.R-project.org/package=polylabelr]

Lackey, L.G., Stein, E.D.: Evaluation of design-based sampling options for monitoring stream and wetland extent and distribution in California. Wetlands **33**, 717–725 (2013)

Mack, J.J.: Ohio Rapid Assessment Method for Wetlands, Manual for Using Version 5.0, Ohio EPA Technical Bulletin Wetland/2001-1-1 edn. Ohio Environmental Protection Agency, Division of Surface Water, 401 Wetland Ecology Unit, Columbus, OH (2001). Ohio Environmental Protection Agency, Division of Surface Water, 401 Wetland Ecology Unit. http://www.epa.ohio.gov/dsw/401/ecology.aspx

Maynard, D.G., Curran, M.P.: Bulk density measurement in forest soils. In: Carter, M.R., Gregorich, E.G. (eds.) Soil Sampling and Methods of Analysis, pp. 863–869. CRC Press, Taylor and Francis Group, Boca Raton, FL, USA (2007). Chap. 66

Millar, J.B.: Wetland classification in western Canada: A guide to marshes and shallow open water wetlands in the grasslands and parklands of the prairie provinces. Report series number 37, Canadian Wildlife Service, Environment Canada (1976)

Mehler, K., Schönin, I., Berli, M.: The importance of rock fragment density for the calculation of soil bulk density and soil organic carbon stocks. Soil Science Society of America Journal **78**, 1186–1191 (2014) https://doi.org/10.2136/sssaj2013.11.0480

Olsen, A.R., Kincaid, T.M., Kentula, M.E., Weber, M.H.: Survey design to assess condition of wetlands in the United States. Environmental Monitoring and Assessment **191**, 268 (2019)

Pearson, T.R.H., Brown, S.L., Birdsey, R.A.: Measurement guidelines for the sequestration of forest carbon. General Technical Report NRS-18, United State Department of Agriculture, Forest Service, Northern Research Station (2007)

Poeplau, C., Vos, C., Don, A.: Soil organic stocks are systematically overestimated by misuse of the parameters bulk density and rock fragment content. SOIL **3**, 61–66 (2017) https://doi.org/10.5194/soil-3-61-2017

Reinecke, M.K., Brown, C.A., Esler, K.J., King, J.M., Kleynhans, M.T., Kidd, M.: Links between lateral vegetation zones and river flow. Wetlands **35**, 473–486 (2015)

Riutta, T., Clack, H., Crockatt, M., Slade, E.M.: Landscape-scale implications of the edge effect on soil fauna activity in a temperate forest. Ecosystems **19**, 534–544 (2016) https://

doi.org/10.1007/s10021-015-9939-9

Ramsfield, T., Shay, P.-E., Trofymow, T., Myrholm, C., Tomm, B., Gagné, P., Bérubé, J.: Distance from the forest edge influences soil fungal communities colonizing a reclaimed soil borrow site in boreal mixedwood forest. Forests **11**, 427 (2020) https://doi.org/10.3390/f11040427

Ruwanza, S.: The edge effect on plant diversity and soil properties in abandoned fields targeted for ecological restoration. Sustainability **11**, 140 (2019) https://doi.org/10.3390/su11010140

Römkens, M.J.M., Young, R.A., Poesen, J.W.A., McCool, D.K., El-Swaify, S.A., Bradford, J.M.: Chapter 3. Soil Erodibility Factor (K). In: Renard, K.G., Foster, G.R., Weesies, G.A., McCool, D.K. (eds.) Predicting soil erosion by water: a guide to conservation planning with the revised universal soil loss equation (RUSLE). Agricultural Handbook Number 703, pp. 65–99. U.S. Department of Agriculture, Washington DC, USA (1997)

Schröder, T., Fleig, F.D.: Spatial patterns and edge effects on soil organic matter and nutrients in a forest fragment of southern Brazil. Soil Research **55**, 649–656 (2017) https://doi.org/10.1071/SR16186

Stevens, J. D L, Jensen, S.F.: Sample design, execution, and analysis for wetland assessment. Wetlands **27**, 515–523 (2007)

Stewart, R.E., Kantrud, H.A.: Classification of natural ponds and lakes in the glaciated prairie region. Resource publication 92, Northern Prairie Wildlife Research Center, Bureau of Sport Fisheries and Wildlife, United States Department of the Interior, Washington (1971)

Stevens, J. D L, Olsen, A.R.: Spatially restricted surveys over time for aquatic resource. Journal of Agricultural, Biological, and Environmental Statistics **4**, 415–428 (1999)

Stevens, J. D L, Olsen, A.R.: Spatially balanced sampling of natural resources. Journal of the American Statistical Association **99**, 262–278 (2004)

Soil Survey Staff: Keys to Soil Taxonomy, 13th edn. USDA-Natural Resources Conservation Service, Washington, DC (2022). USDA-Natural Resources Conservation Service. http://soils.usda.gov/technical/classification/tax_keys/

Soil Survey Staff: Soil Survey Geographic (SSURGO) Database. Natural Resources Conservation Service, United States Department of Agriculture (2024). https://websoilsurvey.sc.egov.usda.gov/App/WebSoilSurvey.aspx

Throop, H.L., Archer, S.R., Monger, H.C., Waltman, S.: When bulk density methods matter: Implications for estimating soil organic carbon pools in rocky soils. Journal of Arid Environments **77**, 66–71 (2012) https://doi.org/10.1016/j.jaridenv.2011.08.020

USEPA: National Wetland Condition Assessment: 2011 Technical Report, Washington, DC (2016)

Whaley, S.D., Minello, T.J.: The distribution of benthic infauna of a Texas salt marsh in relation to the marsh edge. Wetlands **22**, 753–766 (2002) https://doi.org/10.1672/0277-5212(2002)022[0753:TDOBIO]2.0.CO;2

Wade, A.M., Richter, D.D., Cherkinsky, A., Craft, C.B., Heine, P.R.: Limited carbon contents of centuries old soils forming in legacy sediment. Geomorphology **354**, 107018 (2020) https://doi.org/10.1016/j.geomorph.2019.107018
